# Using hyperspectral analysis as a potential high throughput phenotyping tool in GWAS for protein content of rice quality

**DOI:** 10.1186/s13007-019-0432-x

**Published:** 2019-05-23

**Authors:** Dawei Sun, Haiyan Cen, Haiyong Weng, Liang Wan, Alwaseela Abdalla, Ahmed Islam El-Manawy, Yueming Zhu, Nan Zhao, Haowei Fu, Juan Tang, Xiaolong Li, Hongkun Zheng, Qingyao Shu, Fei Liu, Yong He

**Affiliations:** 1 College of Biosystems Engineering and Food Science, Zhejiang University, Hangzhou, 310058 People’s Republic of China; 2 Key Laboratory of Spectroscopy Sensing, Ministry of Agriculture and Rural Affairs, Hangzhou, 310058 People’s Republic of China; 3 State Key Laboratory of Modern Optical Instrumentation, Zhejiang University, Hangzhou, 310058 People’s Republic of China; 4 Jiaxing Academy of Agricultural Sciences, Jiaxing, 314016 China; 5 State Key Laboratory of Rice Biology, Institution of Crop Science, Zhejiang University, Hangzhou, 310058 China; 6 Biomarker Technologies Corporation, Beijing, 101300 China

**Keywords:** Genome-wide association study (GWAS), Spectrology, Protein content, Phenotyping, Rice (*Oryza sativa*)

## Abstract

**Background:**

The advances of hyperspectral technology provide a new analytic means to decrease the gap of phenomics and genomics caused by the fast development of plant genomics with the next generation sequencing technology. Through hyperspectral technology, it is possible to phenotype the biochemical attributes of rice seeds and use the data for GWAS.

**Results:**

The results of correlation analysis indicated that Normalized Difference Spectral Index (NDSI) had high correlation with protein content (PC) with R_NDSI_^2^ = 0.68. Based on GWAS analysis using all the traits, NDSI was able to identify the same SNP loci as rice protein content that was measured by traditional methods. In total, hyperspectral trait NDSI identified all the 43 genes that were identified by biochemical trait PC. NDSI identified 1 extra SNP marker on chromosome 1, which annotated extra 22 genes that were not identified by PC. Kegg annotation results showed that traits NDSI annotated 3 pathways that are exactly the same as PC. The cysteine and methionine metabolic pathway identified by both NDSI and PC was reported important for biosynthesis and metabolism of some of amino acids/protein in rice seeds.

**Conclusion:**

This study combined hyperspectral technology and GWAS analysis to dissect PC of rice seeds, which was high throughput and proven to be able to apply to GWAS as a new phenotyping tool. It provided a new means to phenotype one of the important biochemical traits for the determination of rice quality that could be used for genetic studies.

**Electronic supplementary material:**

The online version of this article (10.1186/s13007-019-0432-x) contains supplementary material, which is available to authorized users.

## Background

Cultivated rice (*Oryza sativa L.*) is one of the major staple food in the world feeding over half of the world’s population [1]. It is becoming more challenging for crop science to feed the world’s fast growing population, which is expected to be over 9 billion by 2050, under the condition of less land, water and more fluctuating climate conditions [2, 3]. Enhancing major crop quality through breeding programs, such as breeding elite varieties with increased yield, improved nutrition and strengthened resistance, is critical for a sufficient, reliable and sustainable world food supply [4, 5]. Therefore, to identify and characterize genes related to these important traits is important for understanding the genetic basis that causes the different phenotypes and breeding modern rice cultivars with higher yield, stress/disease resistance and rice qualities.

The advance of next generation sequencing (NGS) technologies has greatly improved the speed and accuracy of resequencing a large number of genomes, which facilitated the study of rice functional genomics and molecular breeding [6, 7]. In addition, Genome-wide association study (GWAS) is becoming a powerful tool to bridge genotyping and phenotyping, since it rapidly identifies genes associated with phenotypic traits based on SNP markers, which has been applied extensively to dissect rice phenotypic traits [8, 9]. However, there is still a huge gap between phenotyping and genotyping, because the development of phenotyping is much slower compared to genotyping. There are mainly three reasons: Firstly, acquiring traditional agronomic traits is time-consuming and labor-intensive [10]. Secondly, these traits are biased due to a lack of international standards for the phenotypic trait measurement [11]. Thirdly, it is challenging to obtain high-quality phenotypic data, select suitable population size and study the extent of linkage disequilibrium (LD), as long as low structured populations are provided [12, 13]. Rice has different phenotypic traits, such as physical and bio-chemical characteristics, growth performances and biotic/abiotic stress tolerance [13–15]. Many agronomic traits have been applied to investigate rice phenotypes, including physical characteristics such as size, color, shape and texture, or the biochemical attributes such as protein, starch, gel consistency, and aroma [16]. Traditionally, phenotyping methods for rice varieties, like High Performance Liquid Chromatography (HPLC), Gas Chromatography–Mass Spectrometer (GC–MS), and other biochemical processes provided by the national rice identification facilities are expensive, time consuming and labor intensive [17–19]. As a consequence, these methods can only be applied to a small number of samples unless a great amount of labor, time and funding is invested, which has limited the development of the phenotyping process. Therefore, developing a rapid method for high throughput phenotyping is necessary for both phenomics and genomics. Compared with traditional methods of acquiring phenotypic traits, high throughput non-destructive phenotyping is of higher efficiency, accuracy and more standardized. In addition, it is more cost-effective compared with traditional means of acquiring agronomic traits in the long run.

Hyperspectral technology has been greatly applied in plant phenotyping including biochemical attributes such as estimating the canopy water content [20], assessing rice leaf growth [21], determining the rice panicle condition [22] and detecting the severity of damage caused by insects and bacteria [23–25]. It was also applied for evaluating physical characteristics of plants such as the firmness, elasticity, touch resistance of grapes [26]. Feng et al. used a High-throughput hyperspectral imaging system (HHIS) to acquire hyperspectral data for the evaluation of the growth of plants [14]. However, hyperspectral imaging system are usually heavy, large in size, complicated to operate and the data acquired was redundant. Furthermore, the indices they obtained were agronomical traits, including Dry weight, Green leaf area and Chlorophyll content, instead of biochemical attributes of rice quality. The ASD FieldSpec4 Hi-Res spectroradiometer is faster and has wider spectrum range (350–2500 nm), which has been commonly used for soil mineral conditions [27], discriminating different plant species [28], rice crop phenology [29] and biochemical content quantification [30] etc.

Biochemical contents are one essential rice seed parameter that is used for grading rice quality, and it was time-consuming, labor intensive and expensive to measure, so in order to achieve high throughput phenotyping for genetic studies on rice seed quality, such as molecular breeding programs and functional genome study, we attempted to investigate the possibility of using hyperspectral traits extracted from an ASD FieldSpec4 Hi-Res spectroradiometer for GWAS analysis to identify SNP markers and genes that are related to the represented biochemical traits in this research. To our knowledge, there are no published studies that have applied hyperspectral indices of any biochemical contents related to rice quality in GWAS.

In our study, we attempt to extract hyperspectral indices representing biochemical contents that could be used for GWAS analysis, which had never been reported in any published research. The objects of this study were (1) to select hyperspectral indices that could represent the according biochemical trait, (2) using the selected hyperspectral variables as substitutions of biochemical measurements of rice to achieve high-throughput phenotyping for genetic studies and verify the results of GWAS on the extracted hyperspectral indices by comparing the identified SNP markers, Genes and pathways with those of biochemical traits. Eventually through this study, we hope to introduce a possible high throughput plant phenotyping method of biochemical contents that could be used in genetic studies based on hyperspectral technology.

## Methods

### Plant materials

Seeds of eighty cultivated rice accessions (including 56 Japonica type rice and 24 Indica type rice) were grown in an experimental rice field at Rice Breeding Research Station in Jiaxing Academy of Agricultural Sciences, Zhejiang Province, China (N30°50′5″E120°42′59″). The rice field was allocated into 80 rice plots, with each rice variety grown in one rice plot. Rice seeds from five plants of each rice variety, 400 samples in total were collected for 9 phenotypic traits as designed (Table 1). After harvesting, rice seeds were collected and dried by natural air-drying method before being put in labeled paper sample bags and sent to Key Laboratory of Spectroscopy Sensing (KLSS) at Zhejiang University. All rice seed varieties were listed in Additional file 1: Table S1.

**Table 1:** Summary of trait categories

Category	Names	Acronym
Biochemical traits	Amylose content (%)	AC
	Gel consistency	GC
	Crude protein content (%)	PC
	Alkali spreading value	ASV
Hyperspectral traits	Reflectance at wavelength 1177 (nm)	R_1177_
	Reflectance at wavelength 1227 (nm)	R_1227_
	Normalized Differential Spectral Index	NDSI
	Differential Spectral Index	DSI
	Simple Ratio Index	SRI

### Biochemical components measurement

**Fig. 1:**
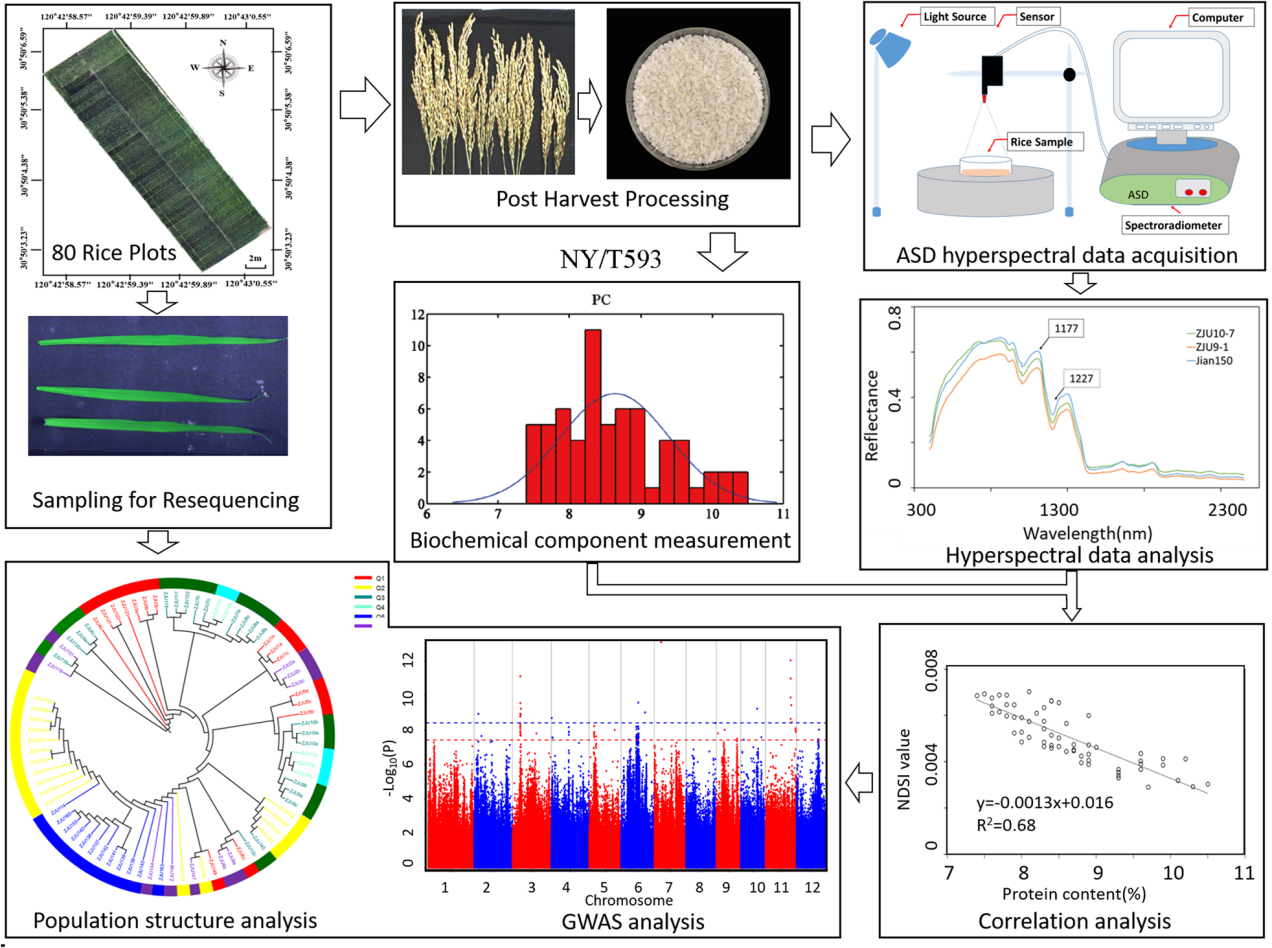
Schematic overview of the primary procedures of extracting hyperspectral traits for GWAS

Air-dried rice seeds were sent to China National Rice Research Institute (CNRRI), Zhejiang province, China, to measure 4 biochemical components that were important for rice quality determination basted on national rice standards NY/T593, including Crude Protein Content (PC), Amylose Content (AC), Gel consistency (GC) and Alkali Spreading Value (ASV) (Table 1). The detailed description of the analytical workflow chart for this study was summarized in Fig. 1. In order to verify the validation of the dataset during statistical analysis, the following criterion was applied: if the data was not normally distributed, the dataset was not accepted. The biochemical components that didn’t meet the aforementioned criterion will not be used for further correlation analysis. Eventually, the traits with an acceptable coefficient covariant and normalized distribution were subjected to hyperspectral trait extraction and Pearson correlation analysis for GWAS analysis.

### Hyperspectral data acquisition

An ASD FieldSpec4 Hi-Res Spectroradiometer (Serial number 18577, Analytical Spectral Devices, Inc., Boulder, Colorado, USA) with a range of 350–2500 nm was used to acquire hyperspectral data. The spectral resolution is 3 nm @ 700 nm, 8 nm @ 1400/2100 nm with sampling intervals (bandwidths) of 1.4 nm @ 350–1000 nm 1.1 nm @ 1001–2500 nm. Rice grains were put in a petri dish (diameter: 8 cm). The sensor of spectroradiometer was fixed vertically down to the opposite to the center of the sample by a detachable rack system and the height between the spectroradiometer sensor and rice sample in petri dish was 20.7 cm. The light source was a 50 W halogenate lamp, which was 27.4 cm in height and 60 degree from the sample surface. The details of the hyperspectral data acquisition system and the spectral data acquisition process are shown in Additional file 2: Fig. S1. For each rice variety, rice seeds of 5 plants were collected and pooled together. Around 3 grams of rice seeds were used to collected hyperspectral data. Each sample was manually loaded to acquire spectral data by spectroradiometer. The reflectance of all rice seeds from each sample that were within the vision field of spectroradiometer was acquired during each reading. For each sample, the spectral acquisition process was repeated 3 times by the spectroradiometer automatically, before they were averaged to represent the sample’s mean spectral reflectance. The data was analyzed on the Matlab software 2014a platform (Matlab works, USA).

### Hyperspectral data process and analysis

The hyperspectral data process followed published protocols with minor changes [31–33]. The acquired hyperspectral data was calibrated by the following equation:

1 $$I_{cal} = \frac{{I_{raw} - I_{dark} }}{{I_{white} - I_{dark} }}$$

Here, *I*_*cal*_, *I*_*raw*_, *I*_*white*_ and *I*_*dark*_ represented calibrated reflectance intensity, original intensity, white reference intensity and dark current, respectively. *I*_*dark*_ was collected by the spectroradiometer automatically; *I*_*white*_ was measured using a white Teflon tile with reflectance close to 100%. Data before 400 nm and after 2450 nm was removed due to the low signal-to-noise ratio before they were used for further analysis.

After calibration, the data were applied for the following analysis. The average spectrum of all samples was extracted. Three indices including Simple Ratio Index (SRI), Normalized Difference Spectral Index (NDSI) and Differential Spectral Index (DSI) were induced for the correlation analysis and as a means to select the optimal wavelength combination for biochemical components. These three indices were used because a number of research had reported normalized difference vegetation index derived indices could be used to estimate biochemical components such as protein content of rice based on the hyperspectral data from rice canopy [34–37]. The formulas are listed below:

2 $$SRI = \frac{{R_{a} }}{{R_{b} }}$$

3 $$NDSI = \frac{{R_{a} - R_{b} }}{{R_{a} + R_{b} }}$$

4 $$DSI = R_{a} - R_{b}$$

Here, *R*_*a*_ and *R*_*b*_ stand for reflectance of two different wavelengths in each formula, respectively. The optimal wave lengths were selected by the following procedure. Reflectance of two random wavelengths was used in the aforementioned formulas to calculate the value of SRI, NDSI and DSI respectively, until all the wavelength combinations were applied. The resulting SRI, NDSI and DSI were then subjected to Pearson correlation analysis. The index of the best performance (the highest the determination coefficient) was selected as the hyperspectral trait for the according biochemical component.

### Sampling, re-sequencing and sequencing data process

One fresh leaf from each rice variety was collected for next generation sequencing. Leaf samples were wrapped in aluminum foil and put in liquid nitrogen for 2 h, prior to being stored at − 80 °C overnight before re-sequencing. Sample Re-sequencing was performed on the Illumina HiSeq X Ten platform (Illumina, Inc., San Diego, CA, USA). Raw sequencing data was first processed with software Illumina Casava 1.8. Raw data was filtered for cleaner reads before further analysis. The filtering process includes the deletion of the adapter sequences, reads with over 10% N contents and reads with over 50% nucleotides whose quality score was lower than 10. The quality score of nucleotides was calculated using the following formula:

5 $$Qphred = - 10log10(e)$$

where *e* stands for the sequencing error rate, *Qphred* represents the quality score of nucleotides.

The BWA software [38] was used for the alignment of resulted clean reads. *Oryza sativa*_IRGSP_1.0 was used as the reference genome which is available on the website of National Center for Biotechnology Information (NCBI). The sequencing depth, genome coverage, and other information of each sample were collected through the alignment process, and mutations were called.

### SNP detection and annotation

The detection of SNPs was achieved mainly using GATK [39] software toolkit. Briefly, the main detection process includes: (1) Picard’s Mark Duplicate tool was used to remove duplicates and mask the effects of PCR-duplication of the resulted clean reads. (2) InDel Realignment was performed using GATK. During this process, local re-alignment was performed to correct the errors caused by the insertion deletion during the aliment process. (3) Base recalibration was performed using GATK to calibrate the SNP quality. (4) Variant calling using GATK, mainly includes SNP and InDel. (5) Strict SNP filtration was performed based on the following criteria: SNP cluster filtering (if there were 2 SNPs within 5 bp, they were filtered out), SNP filtering near Indel (SNPs within 5 bp near Indel were filtered out); and adjacent InDel filtering (if two Indels’ distance is less than 10 bp, they were filtered out) [40].

### Phylogenetic analysis and population-structure study

A neighbor-joining tree of all rice accessions was constructed using MEGA5 software [41] based on the SNP markers and neighbor-joining algorithm (p-distance model with 1000 bootstrap). Based on the high-consistent SNP, the population structure of the samples was analyzed using admixture software [42]. A population structure map was constructed with the hypothesized K-value set from 1 to 15. A clustering process was performed, and the clustering results were cross-validated. The optimal clustering number was determined according to the minimum value of the cross-validation error rate. Based on SNPs, EIGENSOFT software [43] was used to perform Principal components analysis (PCA) analysis to cluster samples based on the first three principal components. The SPAGeDi software [44] was used to estimate the relative kinship of populations. Linkage disequilibrium analysis was performed using Plink2 software [45] to calculate the linkage disequilibrium between SNPs within a distance of 1000 kb on the same chromosome.

### Genome-wide association study (GWAS)

The resulted SNPs (minor allele frequency (MAF) ≥ 0.05) was used for the following GWAS with the selected biochemical and hyperspectral traits. The GWAS analysis was performed using the mixed linear model (MLM) of TASSEL software [46]. The formula for the mixed linear model is as follows:

6 $$y = X\alpha + Q\beta + K\mu + e$$

Here, *Q* stands for the population structure calculated using admixture software, *K* stands for kinship of samples from SPAGeDi software. *X* is the genotype while *y* stands for phenotype. The genome-wide significance thresholds of all tested traits were evaluated with Bonferroni correction:

7 $$P = \frac{0.01}{n}$$

Here, *n* stands for the effective number of independent SNPs. Bonferroni correction was applied here to control the type I error genome-wide. The *P* value threshold for significance in the *Oryza sativa* population was set to be p = 3.788e−09 and p = 3.788e−09 (suggestive and significant, respectively) for the studied population. In this study, only the associations that exceeded the significant *P*-*value* threshold were considered. The extent of local LD was evaluated for each selected significant SNP to determine the interval of each locus.

### Gene annotation and comparison

Functional annotation analysis of genes in the associated regions was performed using different databases including NCBI non-redundant (NR), The Gene Ontology (GO), Clusters of Orthologous Groups (COG), Kyoto Encyclopedia of Genes and Genomes (KEGG) Pathway analysis. A gene was annotated based on the principle that at least 2 databases provided the annotation description. Further validation of candidate genes was obtained by manually screening based on published research and gene function descriptions of the aforementioned databases.

## Results and discussion

### Biochemical traits acquisition

**Fig. 2:**
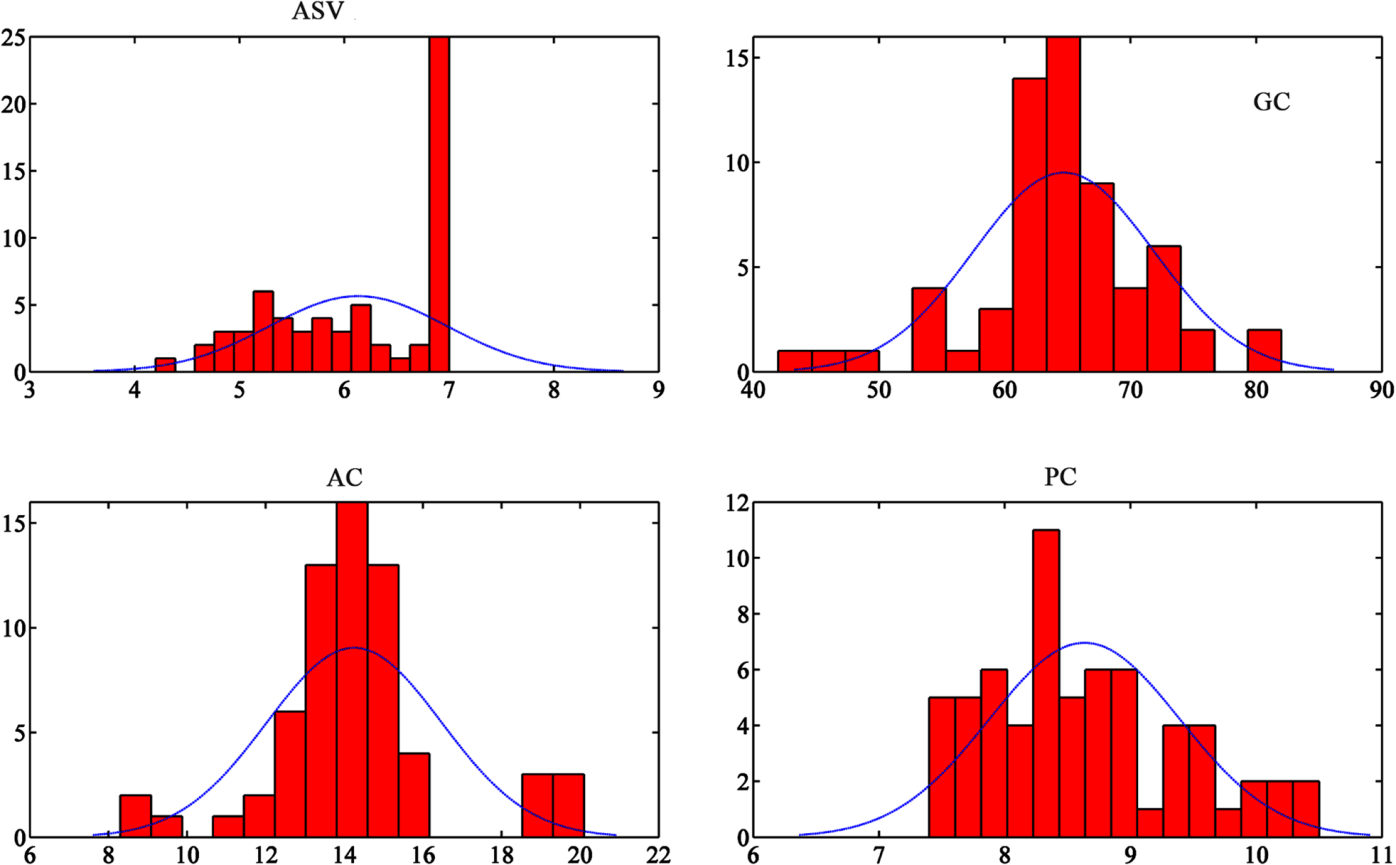
Frequency distribution for all the traits. AC, PC, and GC fit normalized distribution, except for ASV, which is heavily right skewed due to many rice accessions reached the maximum measurement limit

Table 2 shows the summary of the statistics of the phenotypic trait data. Figure 2 shows the frequency distributions of the phenotypic traits. Most of these traits were quantitative and continuous, which suggested that there might be a complex genetic influence, except ASV, which was not continuous (Fig. 2). Based on the general trends of other traits, almost all the other traits were roughly normally distributed.

**Table 2:** Summary statistics for biochemical traits

	Min	Max	Mean	SD	CV
ASV (mm)	3.70	7.00	6.13	0.89	0.15^a^
GC (mm)	42.00	82.00	64.94	6.94	0.11
AC (%)	8.30	20.10	14.20	2.11	0.15^a^
PC (%)	7.40	10.50	8.62	0.77	0.09

^a^CV of ASV and AC are high (≥ 15%)

ASV and AC are two of the multidimensional characteristics relating to rice grain quality that has been used worldwide in rice breeding and process technologies. The degree of degradation test was evaluated visually by inspectors which was graded by a range of scores from 1 to 7. Since a number of the ASV scores of the rice collection reached maximum measurement limit, which was 7, the distribution of ASV value was not normalized and heavily skewed, thus not suitable for Pearson Correlation analysis. Therefore, it was not selected for the following Pearson correlation analysis. ASV is the inverse indicator of the gelatinization temperature (GT) of rice starch granules [47], which is closely related to rice cooking behavior and sensory properties due to crystalline melting and starch solubilisation during cooking process. ASV is also related to AC, therefore, it can be observed in Table 2 that AC has a similar high CV (≥ 15%) as ASV. The CV of ASV and AC were very high (≥ 15%), which means these data sets had a higher dispersion. It could be caused by severe differences between the content of these biochemical traits. A Pearson Correlation analysis was conducted for hyperspectral traits and biochemical traits including PC, AC and GC.

### Hyperspectral data analysis and hyperspectral traits extraction

Overall, the data size of hyperspectral data from the spectrometer was 6.5 MB. Based on the summary of Pearson correlation analysis of GC, AC and PC with all three hyperspectral indices shown in Table 3, GC and AC had low determination coefficient with all three hyperspectral indices (R_SRI_^2^ = 0.21, R_NDSI_^2^ = 0.21 and R_DSI_^2^ = 0.26 for GC; R_SRI_^2^ = 0.24, R_NDSI_^2^ = 0.24 and R_DSI_^2^ = 0.31 for AC), while PC showed high determination coefficient with all three indices, especially NDSI R_NDSI_^2^ = 0.68. Therefore, NDSI was selected for the final GWAS analysis as a hyperspectral trait for PC. The correlation between hyperspectral data and biochemical traits of AC and GC were low (R^2^ < 0.6). This might be because that due to a relatively small sample size and large dispersed data set of AC, the correlation between hyperspectral traits and AC became low. In order to construct a hyperspectral index that could be applied for AC, a bigger sample size would be selected in the future. In addition, because the GC is a complex trait and the nature of the GC data set was not a measurement of the content of a certain chemical/compound, but the length of flow distance [48], which makes it not suitable for hyperspectral technology, because hyperspectral signal was relating to the resonance of certain chemical bonds [25]. There is not any published research on using hyperspectral technology to dissect GC of rice seeds. In addition, it was reported that the accuracy of the algorism developed for the extraction of phenotypic traits using phenotyping platforms is greatly affected by the sample size [49, 50]. At the same level of other effects, as the sample size increased, the degree of freedom of the test increased and the resulting P-value became more significant [51–53].

**Table 3:** Pearson correlation analysis of extracted indices with selected wavelengths

	SRI		NDSI		DSI	
	R^2^	Formula	R^2^	Formula	R^2^	Formula
GC (mm)	0.21	R_1730_/R_1731_	0.21	(R_1731_-R_1730_)/(R_1731_ + R_1730_)	0.26	R_2208_-R_2203_
PC (%)	0.66	R_1227_/R_1177_	0.68	(R_1227_-R_1177_)/(R_1227_ + R_1177_)^a^	0.67	R_1227_-R_1177_
AC (%)	0.24	R_1638_/R_1799_	0.24	(R_1639_-R_1799_)/(R_1639_ + R_1799_)	0.31	R_2028_-R_2002_

^a^NDSI was selected for correlation analysis and GWAS

**Fig. 3:**
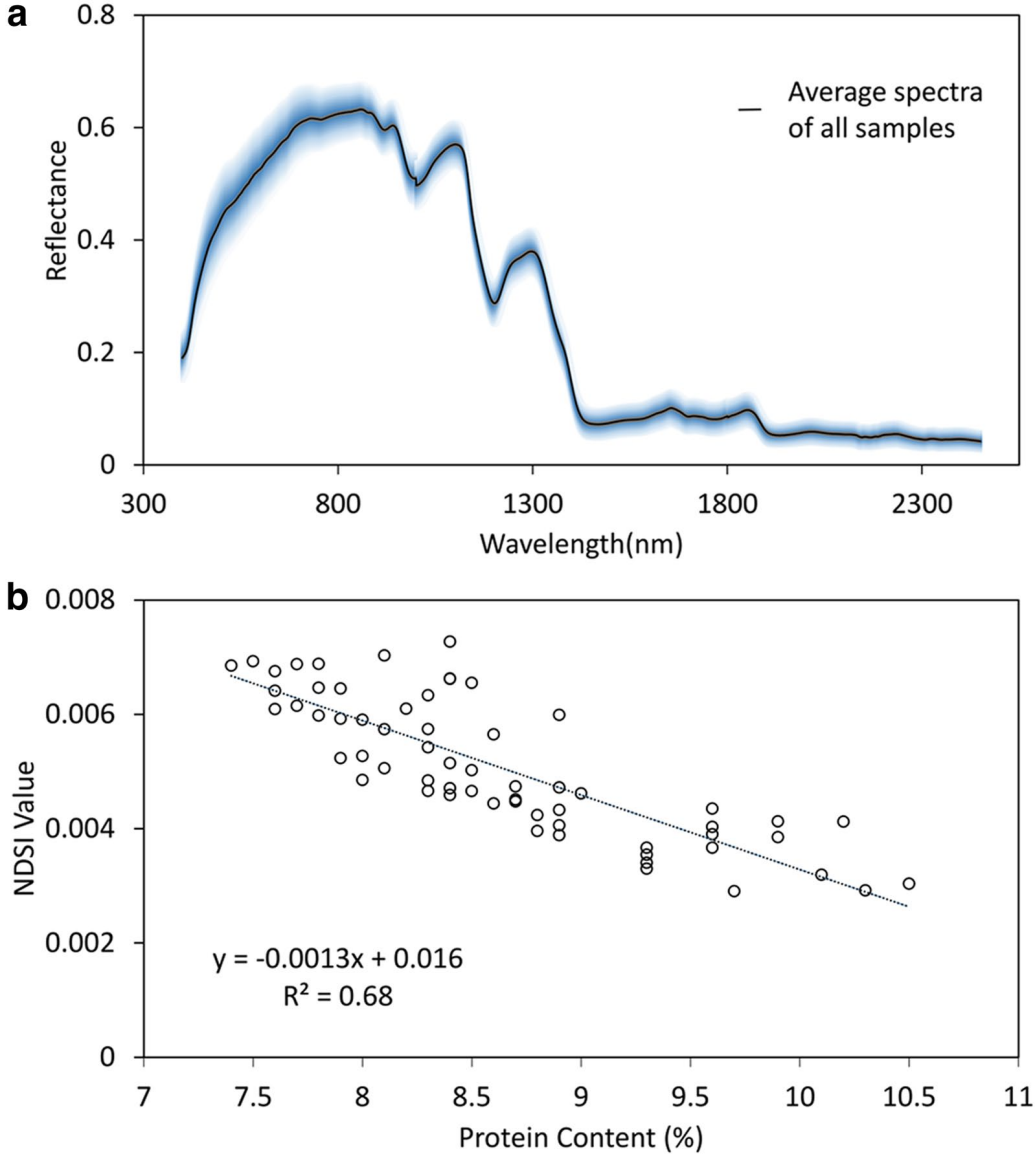
The mean spectra of rice accessions and Pearson correlation analysis. **a** The average spectra of all rice samples. The blue shade indicates 3 times of standard deviation. **b** The correlation analysis of hyperspectral index NDSI and PC used for GWAS, with correlation coefficients of R^2^ = 0.68

Eventually, PC was selected for hyperspectral analysis and GWAS. Two optimal wavelengths were selected for the biochemical trait PC based on formula 3. Figure 3a shows the mean spectra all rice accessions. The results of correlation analysis indicated that NDSI had high correlation with PC (R_NDSI_^2^ = 0.68) (Fig. 3b).

### Genome sequencing and assembly

Sequencing results showed that approximately 12-fold coverage was achieved, generating a total of 419.53 Gbp clean data, with Q30 up to 94.65%. Detailed sequencing data statistics was listed in Additional file 3: Table S2. The average matching ratio between the sample and the reference genome was 98.81%, and the genome coverage was 96.79% (at least one base coverage) when mapped on the reference genome. Only SNP markers with minimum allele frequency (MAF) higher than 0.05 were selected for further analysis. Eventually, a total of 3,398,019 SNP loci and 773,337 Indel loci were identified in this study. The multiple test corrections get more severity as the number of sequencing cases increases. Due to the increase in the depth of sequencing, the marker density is continuously increasing, and the severity of the test is also increasing [51]. Here, the sequencing depth reached 12 × which relatively improved the chance of accurate SNP loci identification. Together with strict SNP and gene screening, filtering and gene function profiling, the resulted SNP/gene loci using hyperspectral traits extracted from hyperspectral data in the GWAS analysis could be more specific and accurate.

### Phylogenetic analysis and population-structure study

**Fig. 4:**
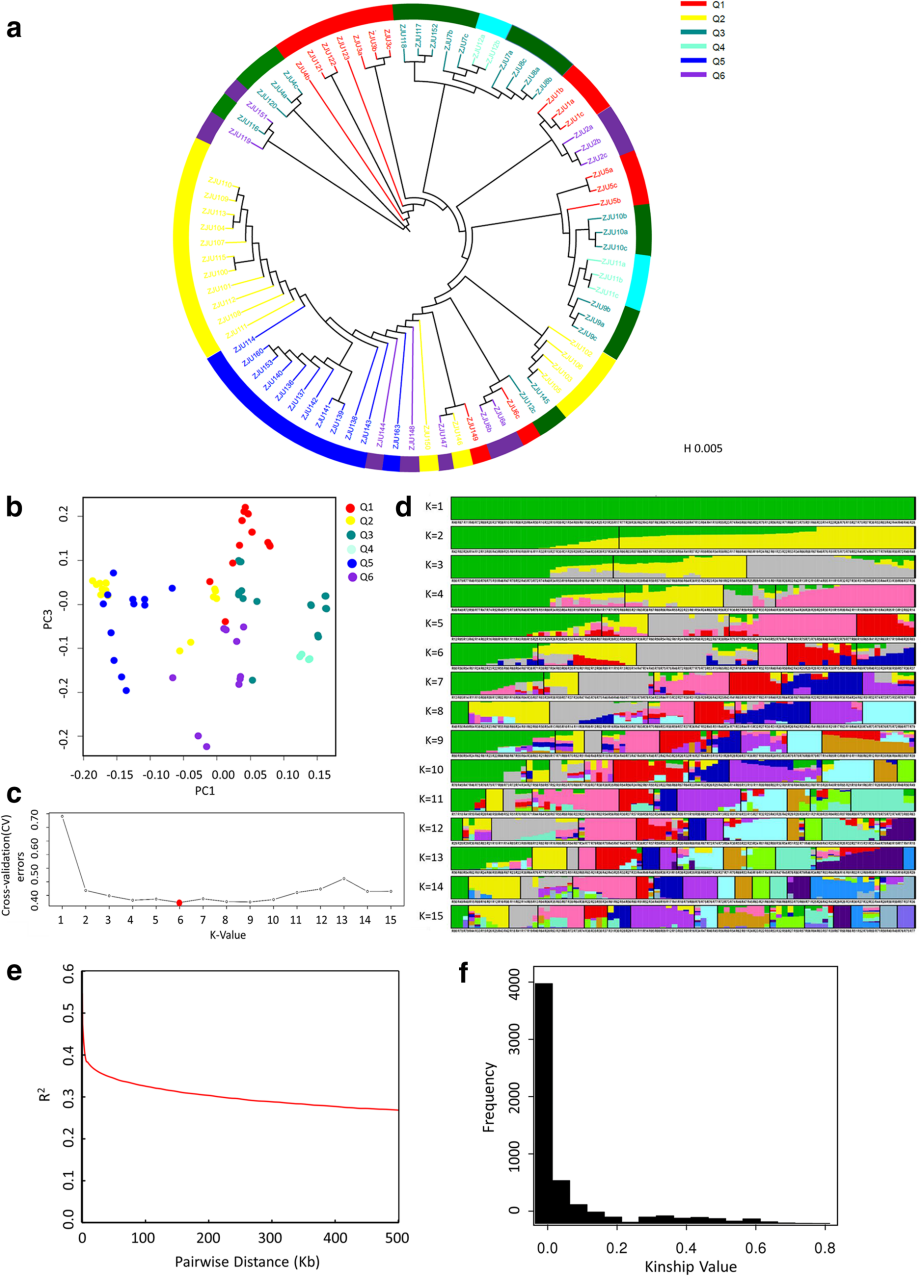
The population structure analysis based on all rice accessions. **a** Neighbor-joining tree of all rice accessions which was constructed from simple matching distance based on whole-genome SNP studies. Branch and circle block colors indicate different subgroup distributions. **b** Principal component (PCA) analysis plots of first and third components for all rice accessions, using the same colors as in **a**. **c** Cross-validation (CV) errors graph. **d** Population structure based on c (K = 6). **e** Genome-wide average LD decay analysis estimated from all rice accessions. **f** Histogram of kinship

The neighbor-joining tree of all rice varieties is shown in Fig. 4a. Figure 4b shows the result of PCA analysis using 2 principle components PC1 and PC3. No Clear separation of subgroups was observed based on components PC1 and PC3 (Fig. 4b), which might be due to the possible complexity of the studied population and potential gene flow of each rice variety due to natural and artificial selection. Therefore, a population structure analysis using ADMIXTURE software was employed to calculate the optimum subgroups for GWAS analysis [54]. Based on the population structure map (Fig. 4d) and cross-validation (CV) errors, the optimum cluster group number in this population was determined to be 6 (Fig. 4c). For better visualization of each subgroup and their position on the phylogenic tree, each rice variety subgroup on the tree was marked by different colors based on the result of population analysis. The linkage disequilibrium decay distance (LDD) of all samples was shown in Fig. 4e. The LD50, which was the LDD when the pairwise coefficient of determination (r^2^) dropped to half its maximum value, was applied to evaluate the linkage disequilibrium. The longer LD50 indicated a smaller chance of gene recombination [55]. On average, it was observed that LD50 of all the rice samples was longer than 100 kb, which indicated a small chance of LD [56]. However, the difference in LDD between each rice chromosomes suggested complicated gene recombination events within this rice cultivar population (data shown in Additional file 4: Fig. S2). The frequency of kinship-value in all the rice accessions were shown in Fig. 4f. As the kinship value increases, the frequency decreases rapidly, indicating a relatively high complexity of population structure, and relatively less kinship within this population.

### GWAS analysis and candidate gene annotation

**Fig. 5:**
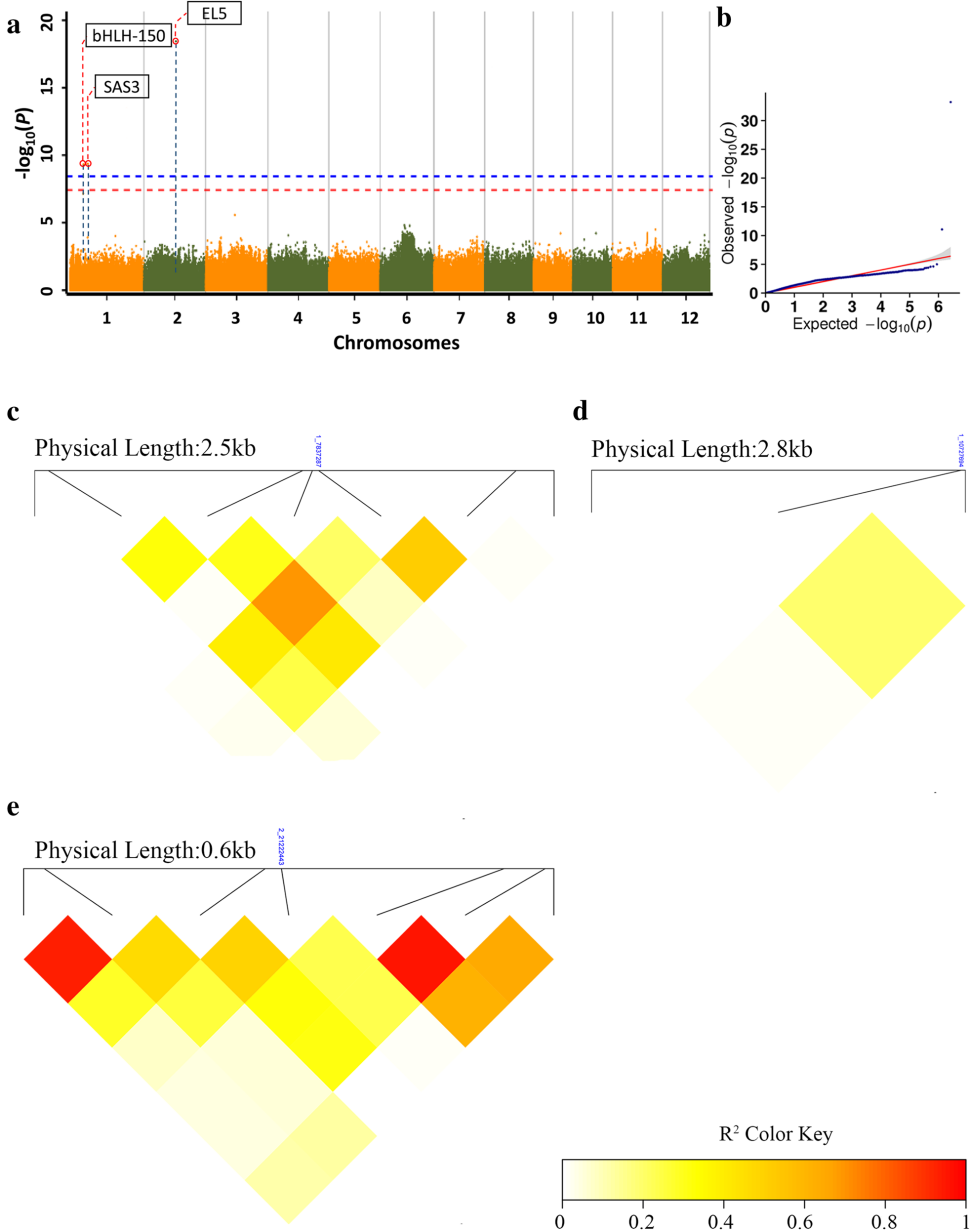
Manhattan plot and QQ plot from genome-wide association studies with local LD block of three selected loci. **a** Manhattan plot by NDSI. **b** Quantile–quantile plot for NDSI. **c** Local LD block of around *Os01g0243400*. The square lattice panel represents the extent of LD based on *r*^*2*^. **d** Local LD block surrounding the locus *Os01g0293000*. **e** Local LD block surrounding the locus Os02g0560200. Blue horizontal dashed line and red horizontal dashed line indicates two genome-wide significance thresholds. The red dashed line is the negative logarithm of the 0.1/SNP number and the blue dashed line is the negative logarithm of the 0.01/SNP number. LD block heat maps (**c**–**e**) were surrounding the peak on chromosome 1, 2 respectively. Navy blue vertical dashed lines indicate the position of SNP loci

The threshold of *p* = 3.788e−09 was used to identify SNPs relating to selected traits PC and NDSI. Manhattan plots and quantile–quantile plots for other traits were shown in Additional file 5: Fig. S2. The summary of identified associated gene numbers for 5 traits were listed in Table 4. The results of hyperspectral trait NDSI and biochemical trait PC in GWAS were compared to evaluate the effects of using hyperspectral traits as phenotyping tool for biochemical traits (Fig. 5). The Manhattan plots and quantile–quantile plots of biochemical attributes of rice crude protein contents represented by PC and NDSI as a substitute of PC in GWAS were shown in Fig. 5.

**Table 4:** A subset of associated loci and candidate genes numbers according to GWAS analysis for PC and NDSI

Trait	Chr.	SNP#	SNP position^a^	P_value	Gene#
NDSI	1	3	7837287	2.75E−10	65
	1		10727694^b^	2.40E−10	
	2		21222443^b^	4.72E−19	
PC	1	2	10727694^b^	8.53E−12	43
	2		21222443^b^	6.09E−34	

^a^Position in bp; Chr. Chromosome

^b^Hyperspectral index NDSI could locate the exact SNP positions as PC

As it was shown in the Manhattan plots and quantile–quantile plots, by biochemical trait PC, we identified top peaks located on chromosomes 1 and 2, which were positions correlating with known loci *SAS3* and EL5, respectively (Fig. 5a, b). These two loci were also detected by hyperspectral index NDSI (Fig. 5b), indicating hyperspectral index NDSI, could be used for GWAS analysis and identifying the all the gene loci as by biochemical trait PC. NDSI index also detected one SNP position 7837287 located on chromosome 1 (Table 4), which was not identified by PC trait. Hu et al. [57], reported that in their study of doubled haploid lines, there were quantitative loci located on chromosome 1. In addition, according to Zhang et al. [58] four protein fractions and crude protein contents were located on chromosome 1 and 2 based on their study on seventy-one recombinant inbred lines, including, Albumin, Globulin, Prolamin, Glutelin and crude protein, which is similar to our results.

SAS3 *(OsSAMS3)* for S-adenosyl-l-methionine synthetases (SAMS) on chromosome 1 was previously recorded to be important for histone H3K4me3 in rice, because it regulates the expression of genes related to flowering development by DNA methylation. By knocking down SAS3, the expression of the flowering key genes including Early heading date 1 (Ehd1), Hd3a and RFT1 (rice FT-like genes) were greatly decreased, which lead to severe late flowering. This might cause decreasing of rice milking stage, which was important for the accumulation of seed nutrition including rice protein content [59]. Meanwhile, rice *EL5* belongs to an ATL family gene, which is characterized by a transmembrane domain at the N-terminal and a RING-H2 finger domain (RFD). Koiwai et al. [60, 61] reported that EL5, function as a membrane-anchored E3, was important for the maintenance of cell viability after root primordial formation was initiated. It was a ubiquitously expressed protein in rice, which could affect rice plant growth status by affect the development of root initiation, thus influence the accumulation of rice nutrition including protein content.

LD block heatmaps based on the LD of each identified SNP loci are shown in Fig. 5c–e. The LD analysis of the three loci showed that these markers had relatively low LD parameter (R^2^ < 0.6) which indicates a relatively low correlation with each other. These LD regions indicated relatively strong inheritability with their traits accordingly, which might not be due to LD block effects.

Potential candidate gene loci that could be related to PC content based on KEGG annotation, GO annotation and NR annotation (NCBI) were summarized in Table 5. These gene loci were identified in a number of studies that have reported functions that might directly or indirectly be related to protein content variation. One research reported that *glx*-*1*, a glyoxalase gene, could express protein OsGlyI, which might be relating to improving abiotic stress tolerance and grain yield in rice [74]. During the past few years, more research has reported the function of non-coding RNAs and histone modifications on the regulation of transcription, flowering periods, rice reproduction, and development of rice seeds. Two-component response regulator *ORR2* was reported to cause rice morphology variation and cytokinin metabolism [70].

**Table 5:** Subset of potential candidate gene loci related to PC

#Gene name	Chr	SNP location	Gene description	References
*Os01g0242600*	1	7837287	C2 domain-containing protein At1g53590	[62–65]
*Os01g0243400*	1	7837287	Transcription factor bHLH150-like	[66, 67]
*Os01g0243700*	1	7837287	Glucan endo-1,3-beta-d-glucosidase	[68]
*Os01g0292900*	1	10727694	Sphingosine-1-phosphate lyase gene *OsSPL1*(SPL1)	[69]
*Os01g0293000*	1	10727694	S-adenosylmethionine synthase 3 (SAS3)	[59, 63]
*Os02g0557800*	2	21222443	Two-component response regulator *ORR2*	[63, 70]
*Os02g0559300*	2	21222443	Cyclin-dependent kinase C-2-like; probable serine/threonine-protein kinase At1g54610	[62, 63]
*Os02g0560200*	2	21222443	E3 ubiquitin-protein ligase EL5 (EL5)	[60, 71–73]
*Os02g0560300*	2	21222443	3-(3-Hydroxy-phenyl)propionate/3-hydroxycinnamic acid hydroxylase-like	

The basic helix-loop-helix (bHLH) proteins, a superfamily of TFs, are one of the largest TF families in plants, which includes 177 *bHLH* genes in the rice genome. These conserved TFs have a diverse variety of functions in many plant signaling processes that regulates the expression of functional proteins involved in different biological processes including cell proliferation and differentiation [75], root development, anthocyanin biosynthesis, plant morphology and fruit pigment accumulation [76], mineral uptake [77], abiotic stress response [78] and seed morphology [79], etc. In the study of pollen development regulation conducted by Ko et al. [64] it was found that *bHLH142* played an important role in pollen development, which could potentially affect the flowering date and the development of grains. According to Liu, the flowering time was associated with rice seed protein content [80]. Most interestingly, one research on the wheat grain storage proteins (GSPs) in response to nitrogen application reported that 26 differentially expressed genes (DEGs) were related to the accumulation of GSPs. In their study, with an increasing level of nitrogen, the GSPs were remarkably increased, while three bHLH genes including TFs *bHLH*-*150* were evidently down-regulated 10–25 and 15–35 days after anthesis [67]. This research suggests the function of *bHLH*-*150* in regulating the accumulation of rice storage protein. It indicates that SNP locus sf017837287 identified by NDSI but not PC is related to rice protein content. To further validate this result, a gene function analysis of *bHLH*-*150* in rice would be conduct in the future studies. However, this further illustrates the promising potential of hyperspectral traits in GWAS study which is rapid to acquire and can identify promising genes while biochemical traits could not. The detailed gene annotations were listed in Additional file 6: Table S3.

### Comparison of gene loci identified by biochemical trait PC and hyperspectral trait NDSI

**Fig. 6:**
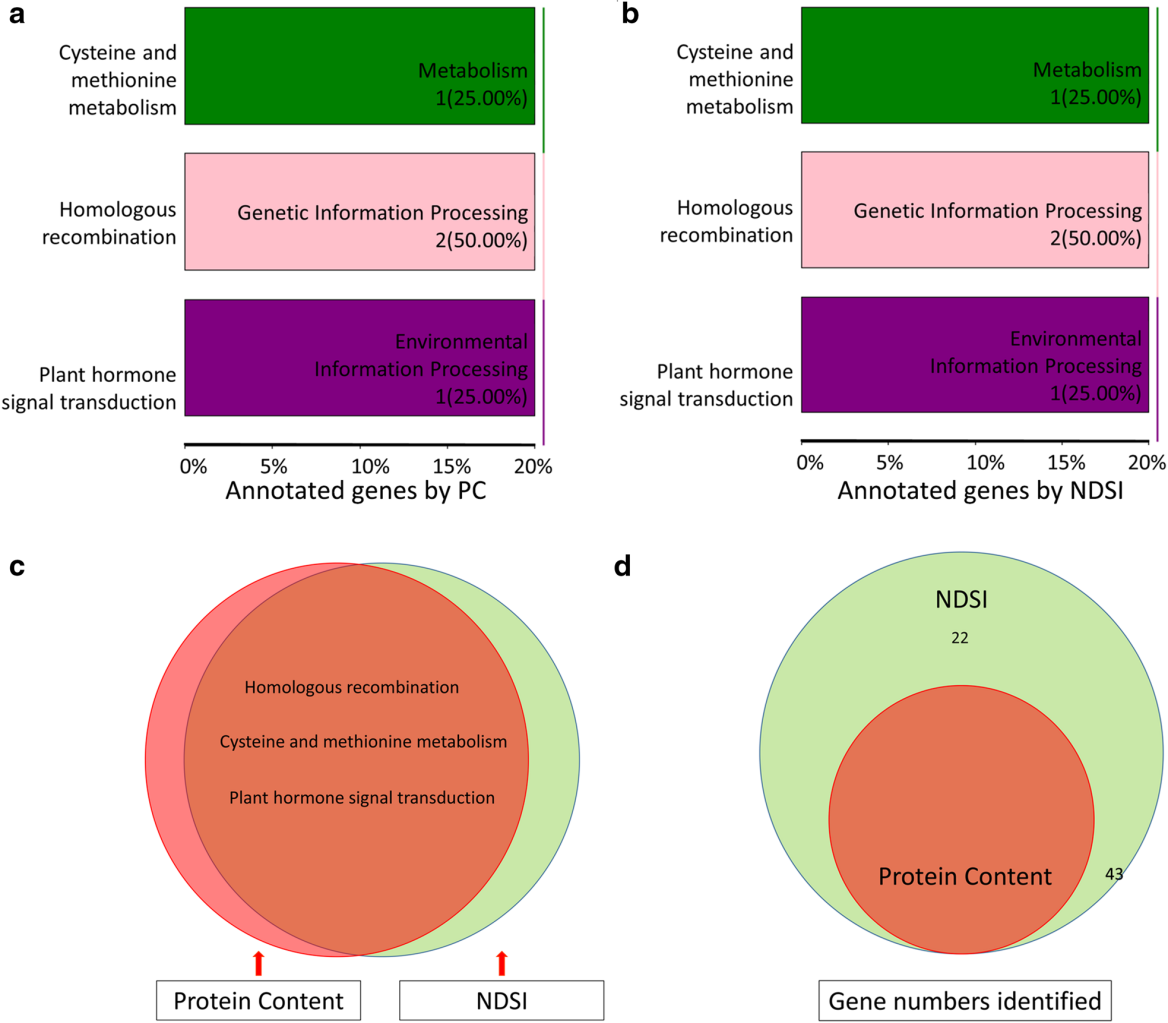
KEGG enrichment histogram and Vernn diagram. **a** Pathway association identified using biochemical trait, PC. **b** Pathway association identified using hyperspectral trait NDSI. **c** Venn diagrams of pathways identified by PC and NDSI. **d** Venn diagrams of genes identified by PC and NDSI

Kegg annotation histogram shows that traits PC (Fig. 6a) and NDSI (Fig. 6b) annotated exactly 3 same pathways including Cysteine and methionine metabolism, homologous recombination and plant hormone signal transduction. These three pathways are included in three biological processes including, metabolism, genetic information processing and environmental information processing. The cysteine and methionine metabolic pathway was reported important for biosynthesis and metabolism of some of amino acids/protein, because results of the reported research showed an increase of cysteine and glutathione, which was accompanied by an increasing level of free methionine and methionine that was incorporated into water-soluble protein fractions in rice seeds. It was noted that there were more isoleucine, leucine, and valine contents in the transgenic lines of rice with high activity of cysteine and methionine metabolic pathway [81, 82]. The top hits of the SNP locus (*Os01g0293000*) in NCBI gene bank was annotated to be related to the biosynthesis of amino acids. Gene function analysis of genes identified by NDSI but not detected by PC on chromosome 1 position 7837287 was able to identify annotated gene *bHLH*-*150* which was reported to be involved in the regulation of grain storage protein, which indicates that NDSI could possibly serve as a substitute of PC and identify more genes than PC involved in rice protein content in GWAS.

The distribution of pathways and gene loci that were identified by PC and NDSI based on Kegg database were summarized in the Venn diagrams in Fig. 6c, d, respectively. It can be observed from Kegg pathway annotation, that NDSI was able to locate the same biological pathway as PC. Meanwhile, in terms of gene numbers, NDSI located 65 genes, which covered all the 43 genes that were identified by PC. This result further proofed the accuracy and feasibility of hyperspectral index NDSI as a hyperspectral trait that could be used in GWAS as a substitute of PC. Since the acquisition of NDSI was cheaper, less biased from manual measurement, accurate in identifying SNPs, and much more rapid, this method is potentially a high throughput phenotyping tool in GWAS for protein content of rice quality.

### Rapid acquisition of biochemical data by hyperspectral technology for genetic studies

Traditionally, it is time-consuming and labor-intensive to acquire most of the biochemical component traits, but using spectroradiometer we not only obtained standardized phenotypic traits accurately but achieved high-through, which could be applied to keep up with the advancing speed of genotyping [2, 10]. Since the gap between the development of phenotyping and genotyping are mainly caused by the lacking of standards as well as low efficiency of current phenotyping method [83]. The study of high throughput phenotyping method using different sensors, computers and integrated platforms could improve the phenotyping speed, the standardization of phenotypic traits, as well as incorporate phenotyping methods/traits that could not be measured or identified by human being [84, 85]. Optimize the criteria for phenotypic measurement and identification systems is of great importance for the advancing of phenotyping. Plant phenotypes are determined by the combination of plant genotypes and different environment conditions where the plant grows. Changes of environment conditions are difficult to control and will cause great interference. Coupled with the phenotypic measurement bias due to human interference, it will reduce the significance of the test. A good solution is to use an automatic phenotype identification system that can effectively reduce the error of manual operation. Strictly following the criteria of processing and selection of phenotypic data set is important during the extraction of feasible hyperspectral indices. In order to get the best results, CVs lower than 0.15 and normal data distribution are applied in this study for the best performance of Pearson correlation analysis and index extraction.

In the future study, more hyperspectral traits representing different biochemical traits, such as starch, components relating to rice fragrance and texture, need to be extracted for GWAS analysis to improve application of the SNP identification process. Meanwhile, more gene function analysis of GWAS on hyperspectral traits are needed to fully address the effect of hyperspectral traits in genetic studies. Other information related to this research is listed in Additional files 7, 8, 9, 10.

## Conclusions

In this study, we tried to apply high through-put spectroradiometer hyperspectral data to extract hyperspectral traits that were highly related to biochemical attributes for rice quality. One resulted hyperspectral trait NDSI was used for GWAS as an alternative for rice protein content to investigate the possibility of using hyperspectral trait for genetic study. The results showed that the application of hyperspectral trait NDSI had the potential to identify the same genes/pathways as PC and in addition, NDSI detected one more SNP locus that is related to grain protein content which could not be identified by PC. In conclusion, hyperspectral traits have the potential to be applied to GWAS as an alternative to traditional time-consuming and labor-intensive biochemical component measurements, which could greatly improve the phenotyping speed and decrease bias caused by human operation for genetic study. This research provides a potential new method to phenotype biochemical traits of rice for genetic studies based on the hyperspectral technology.

## Additional files

**Additional file 1: Table S1.** Information of all rice accessions.

**Additional file 2: Fig. S1.** The setting of the hyperspectral data acquisition system.

**Additional file 3: Table S2.** Summary of re-sequencing results.

**Additional file 4: Fig. S2.** LD decay of all rice accessions.

**Additional file 5: Fig. S3.** GWAS results of other traits.

**Additional file 6: Table S3.** Summary of gene annotation.

**Additional file 7: Table S4.** Measurements of biochemical traits.

**Additional file 8: Table S5.** MAF of all identified SNPs.

**Additional file 9: Fig. S4.** Distribution of reflectance for signal-to-noise ratio.

**Additional file 10: Fig. S5.** The distribution of accumulative SNP depth.

## Data Availability

ll the rice varieties included in the study are available from the corresponding author on reasonable request. All the sequencing raw data were deposited in Sequence Read Archive (SRA) database in a BioProject under the SRA accession ID “PRJNA491628” on The National Center for Biotechnology Information (NCBI). All generated or analyzed data during this study are included in this published article and its supplementary information files. This article is not published nor is under publication elsewhere.

## Abbreviations

GWAS: genome-wide association study
CV: coefficient of variance
STDEV: standard deviation
SNP: single nucleotide polymorphism
MLM: mixed linear model
InDel: insertion or deletion of bases
PCA: principal components analysis
LDD: linkage disequilibrium decay distance
LD: linkage disequilibrium
NCBI: National Center for Biotechnology Information Search database
NR: RefSeq non-redundant proteins
KEGG: Kyoto Encyclopedia of Genes and Genomes
GO: gene ontology
COG: clusters of orthologous groups
GSPs: grain storage proteins
DEGs: differential expressed genes
