# Supplementary material for: Using hyperspectral analysis as a potential high throughput phenotyping tool in GWAS for protein content of rice quality
Source: Plant Methods. 2019 May 23;15:54. doi: 10.1186/s13007-019-0432-x (PMC6532189; doi:10.1186/s13007-019-0432-x)
Supplement: Supplementary file 2 — Additional file 2: Fig. S1. The setting of the hyperspectral data acquisition system. [file 13007_2019_432_MOESM2_ESM.pdf]

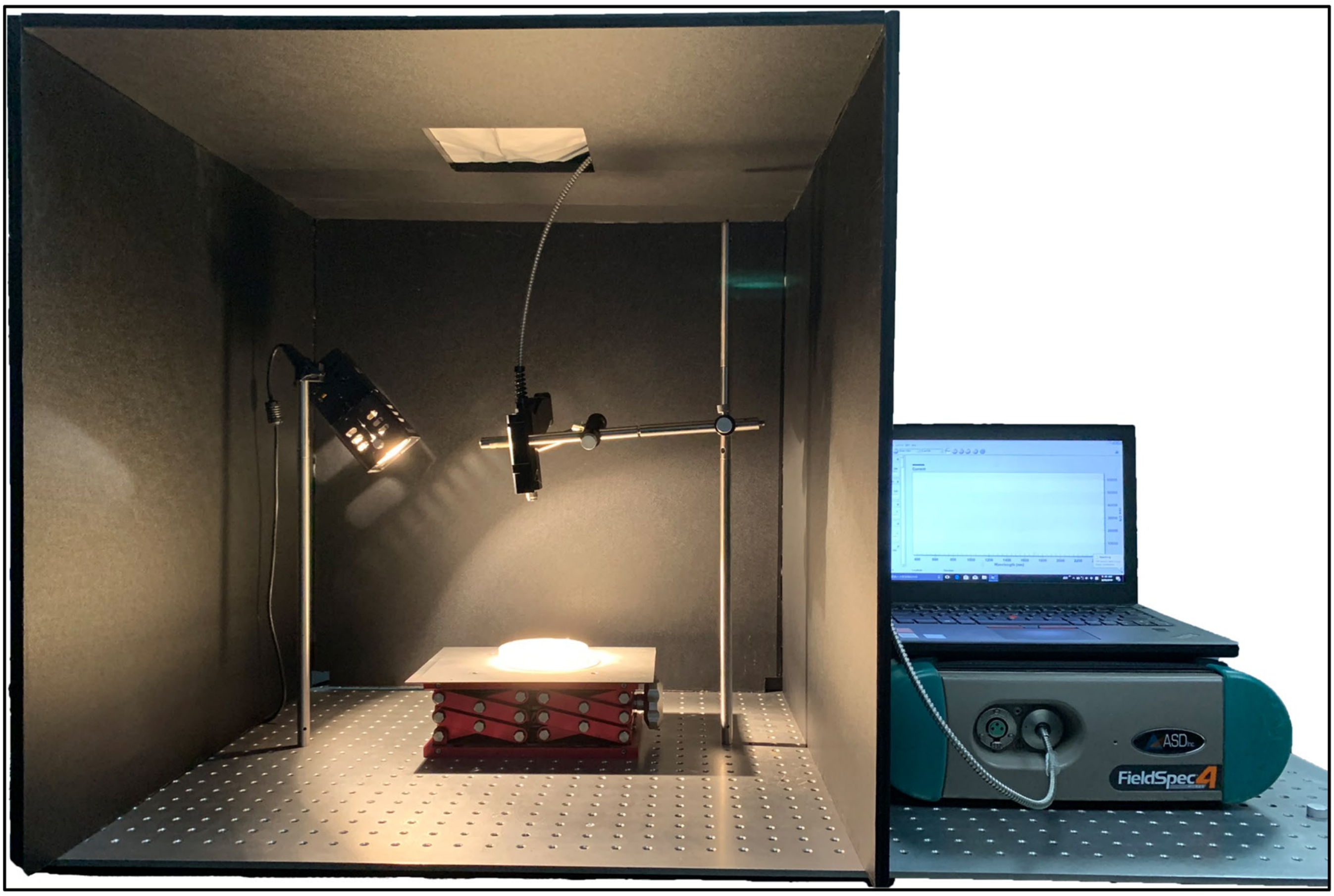

Additional file: Fig.1S

The setting of the hyperspectral data acquisition system

The details of the hyperspectral data acquisition system and the spectral data acquisition process are shown above. For each rice variety, rice seeds of 5 plants were collected and pooled together. Around 3 grams of rice seeds were used to collected hyperspectral data. Each sample was manually loaded to acquire spectral data by spectroradiometer. The reflectance of all rice seeds from each sample that were within the vision field of spectroradiometer was acquired during each reading. For each sample, the spectral acquisition process was repeated 3 times by the spectroradiometer automatically before they were averaged to represent the sample's mean spectral reflectance.
