# Supplementary material for: Using hyperspectral analysis as a potential high throughput phenotyping tool in GWAS for protein content of rice quality
Source: Plant Methods. 2019 May 23;15:54. doi: 10.1186/s13007-019-0432-x (PMC6532189; doi:10.1186/s13007-019-0432-x)
Supplement: Supplementary file 4 — Additional file 4: Fig. S2. LD decay of all rice accessions. [file 13007_2019_432_MOESM4_ESM.pdf]

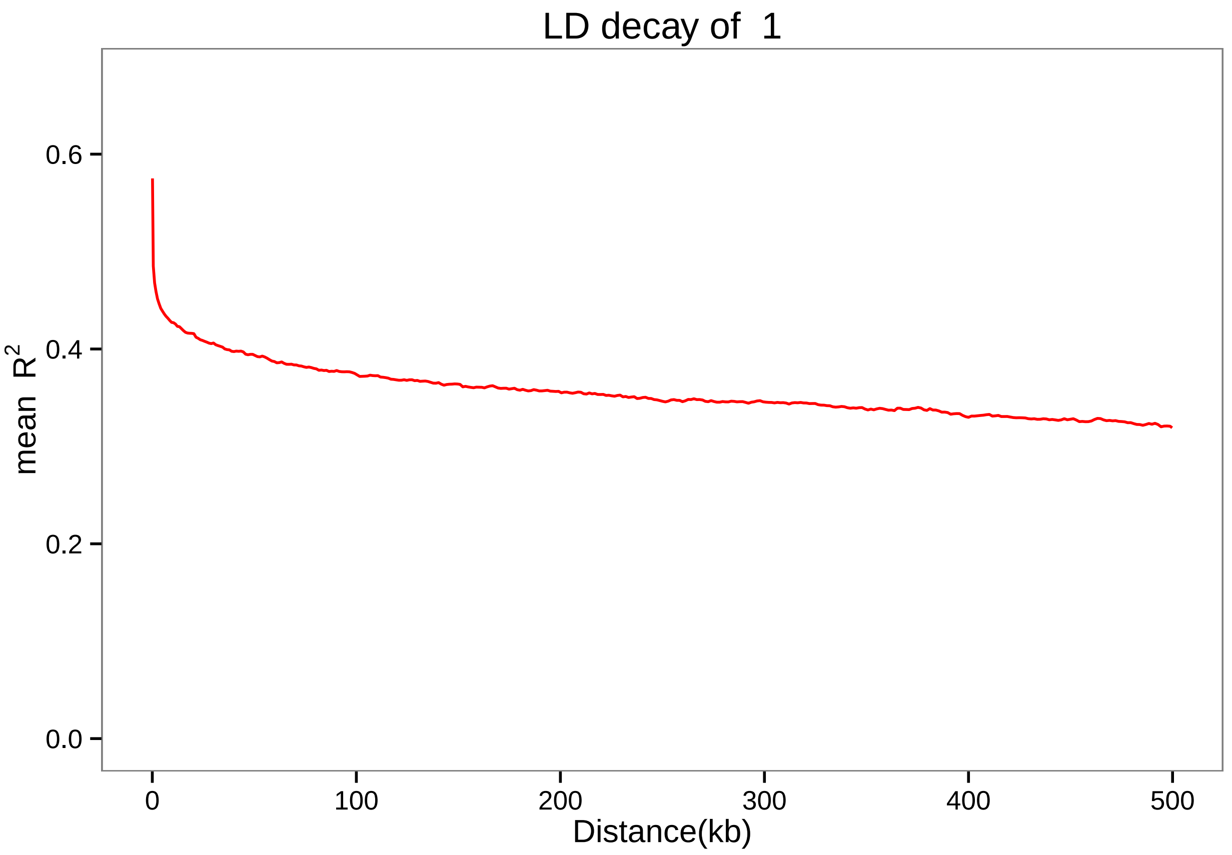
LD decay of 1
mean R2
0.0
0.2
0.4
0.6
0
100
200
300
400
500
Distance(kb)

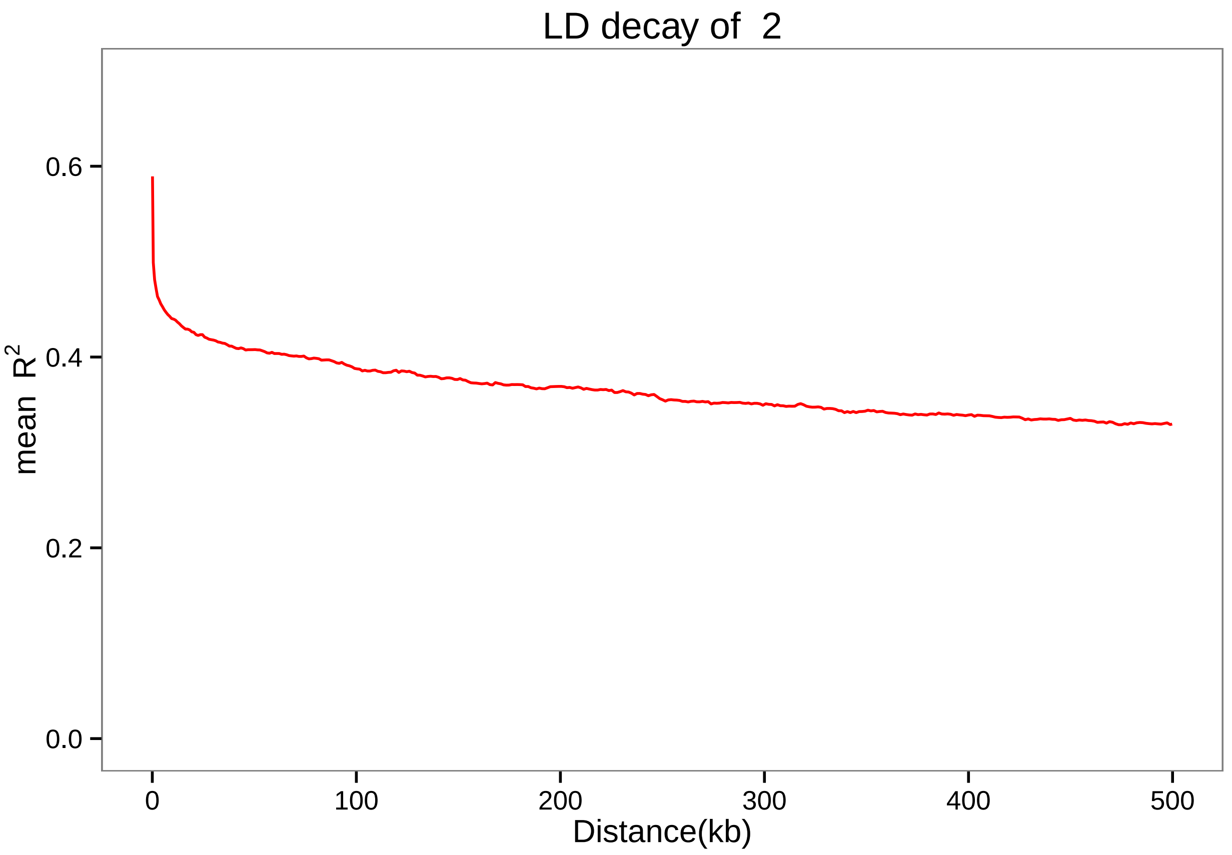
LD decay of 2
mean R^2
0.0
0.2
0.4
0.6
0
100
200
300
400
500
Distance(kb)

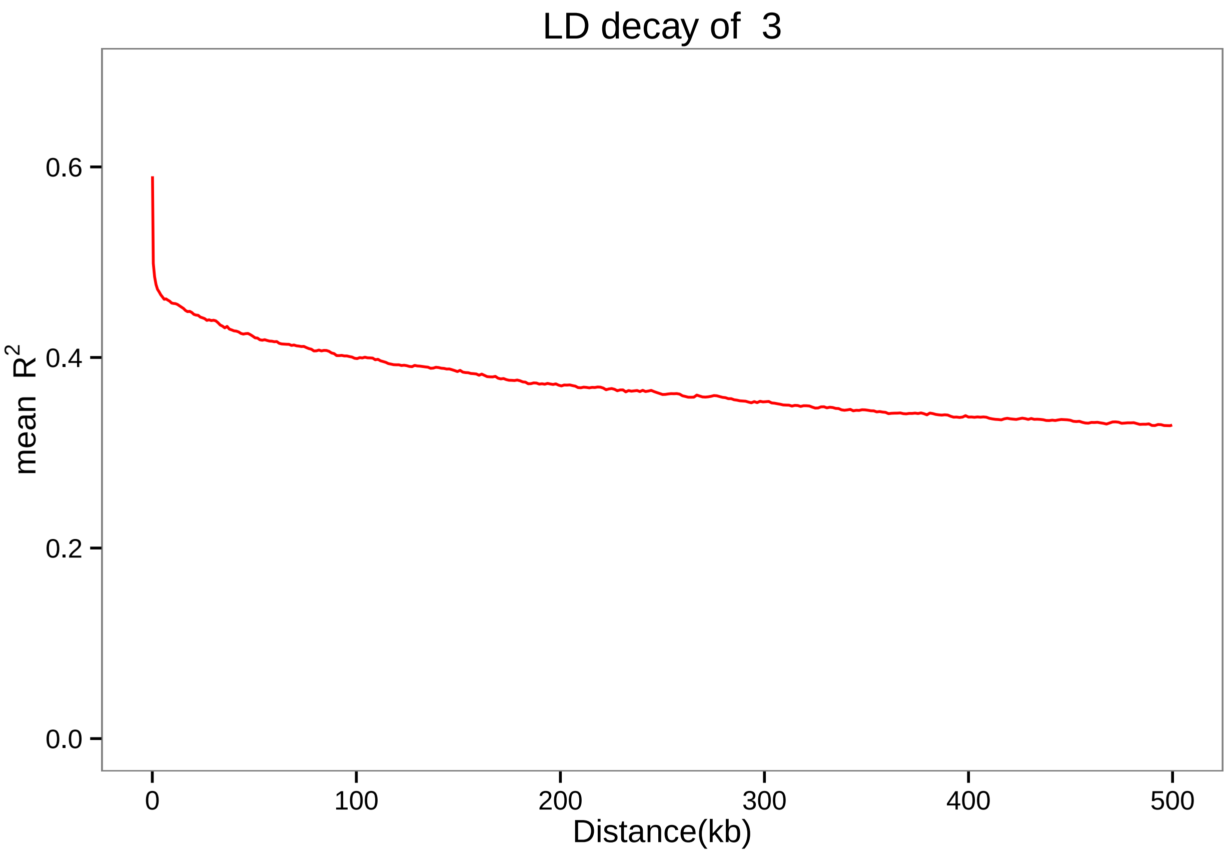

LD decay of 3
0.0
0.2
0.4
0.6
mean R^2
0
100
200
300
400
500
Distance(kb)

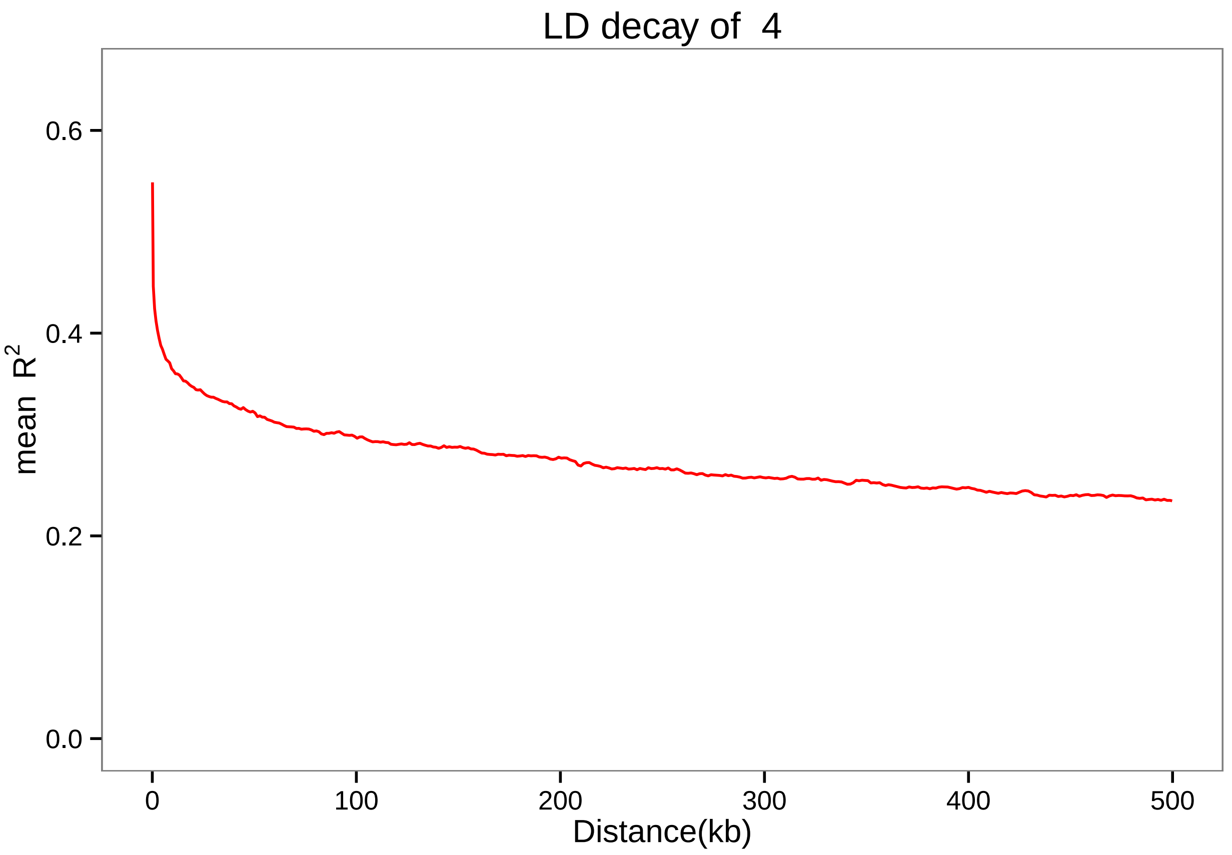

LD decay of 4
0.6
0.4
0.2
0.0
mean R2
0
100
200
300
400
500
Distance(kb)

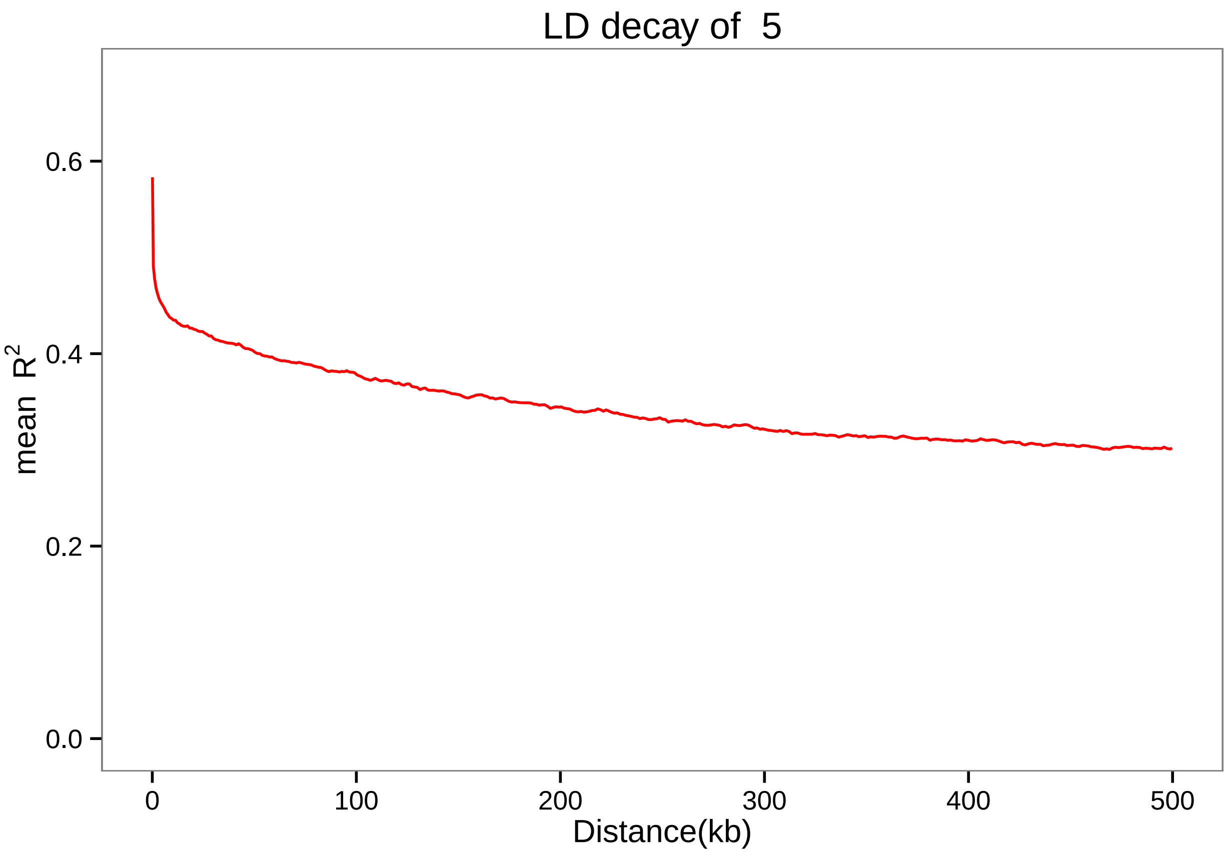

LD decay of 5
mean R^2
0.6
0.4
0.2
0.0
0
100
200
300
400
500
Distance(kb)

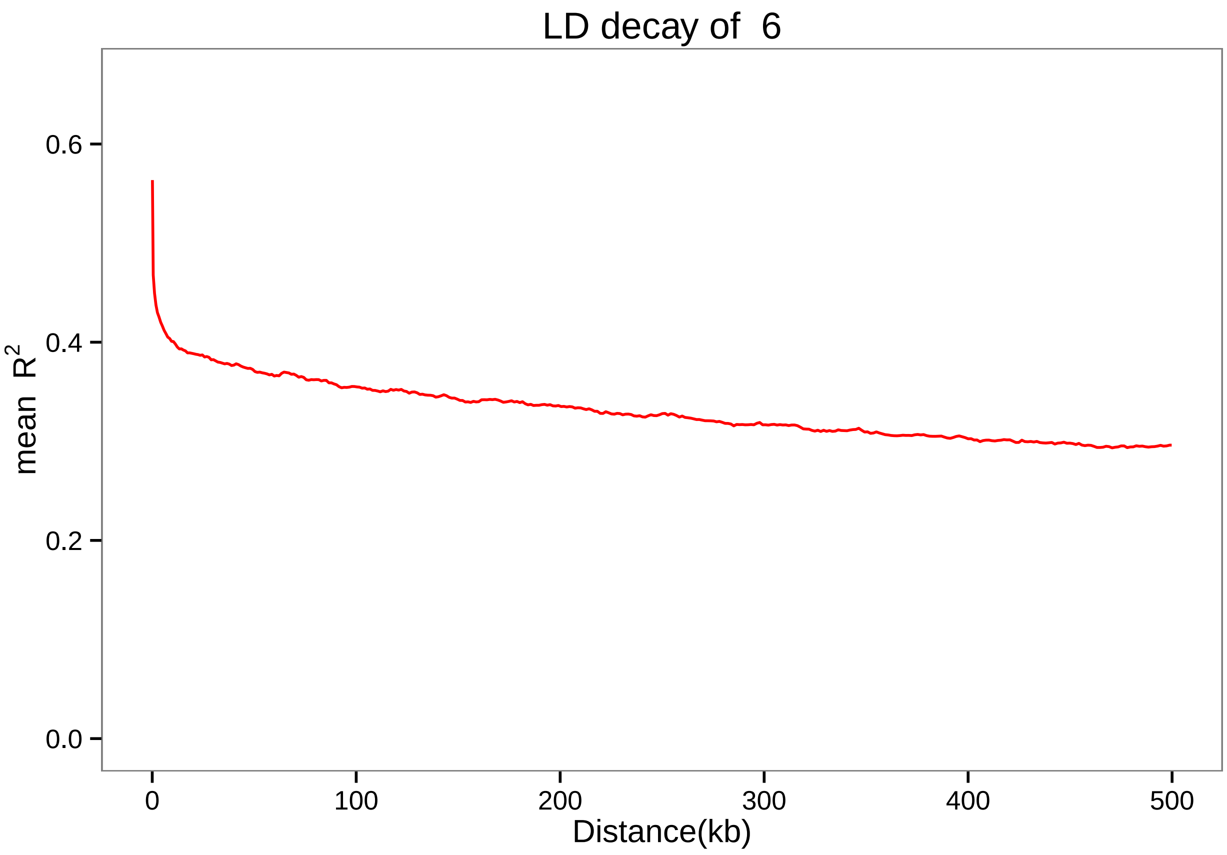
LD decay of 6
0.6
0.4
0.2
0.0
mean R^2
0
100
200
300
400
500
Distance(kb)

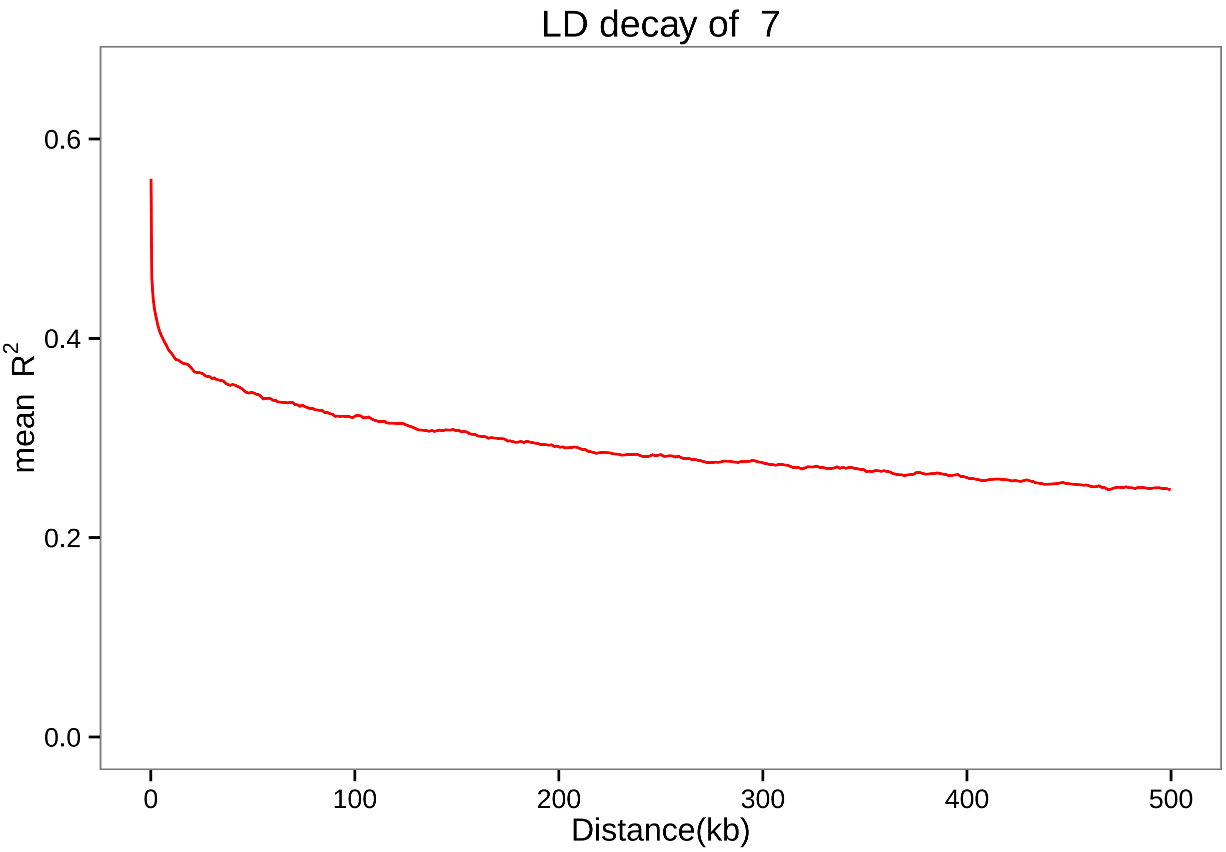
LD decay of 7
mean R2
0.6
0.4
0.2
0.0
0
100
200
300
400
500
Distance(kb)

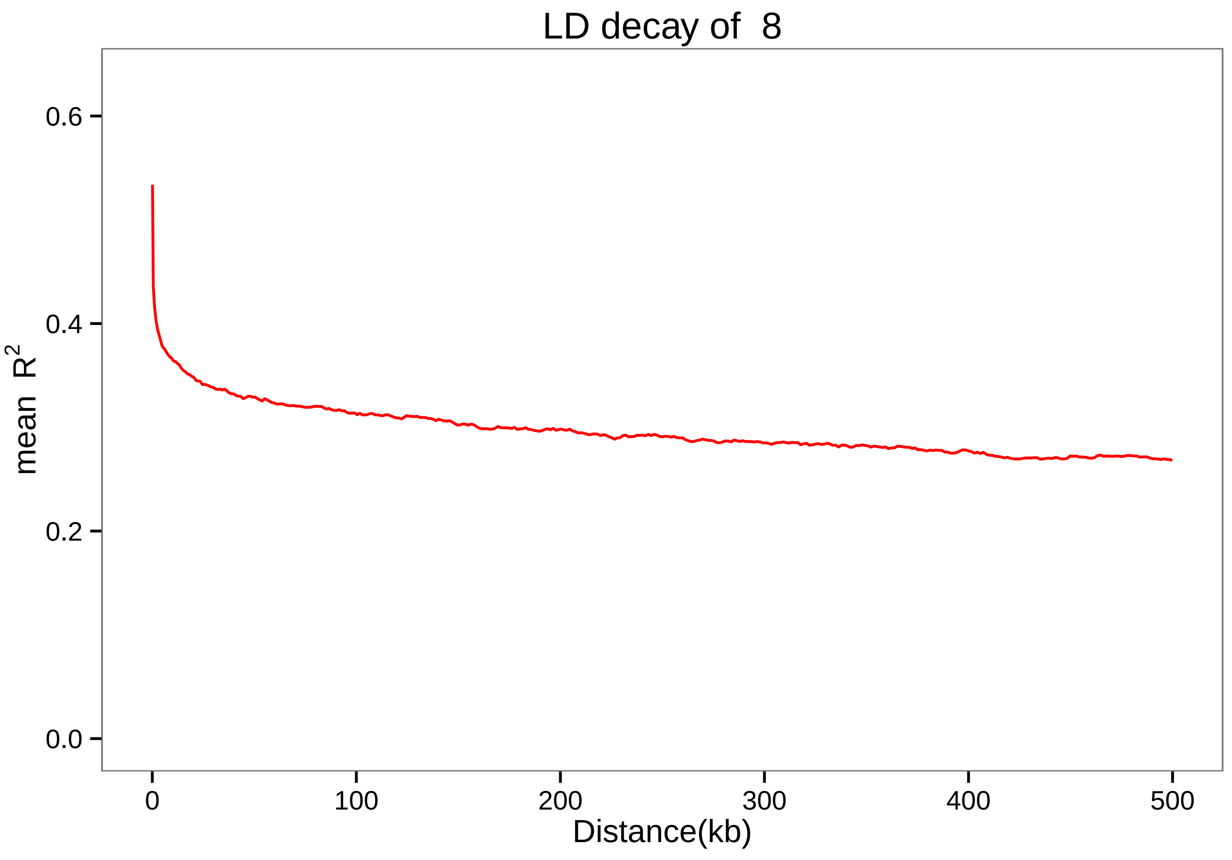

LD decay of 8
mean R^2
0.6
0.4
0.2
0.0
0
100
200
300
400
500
Distance(kb)

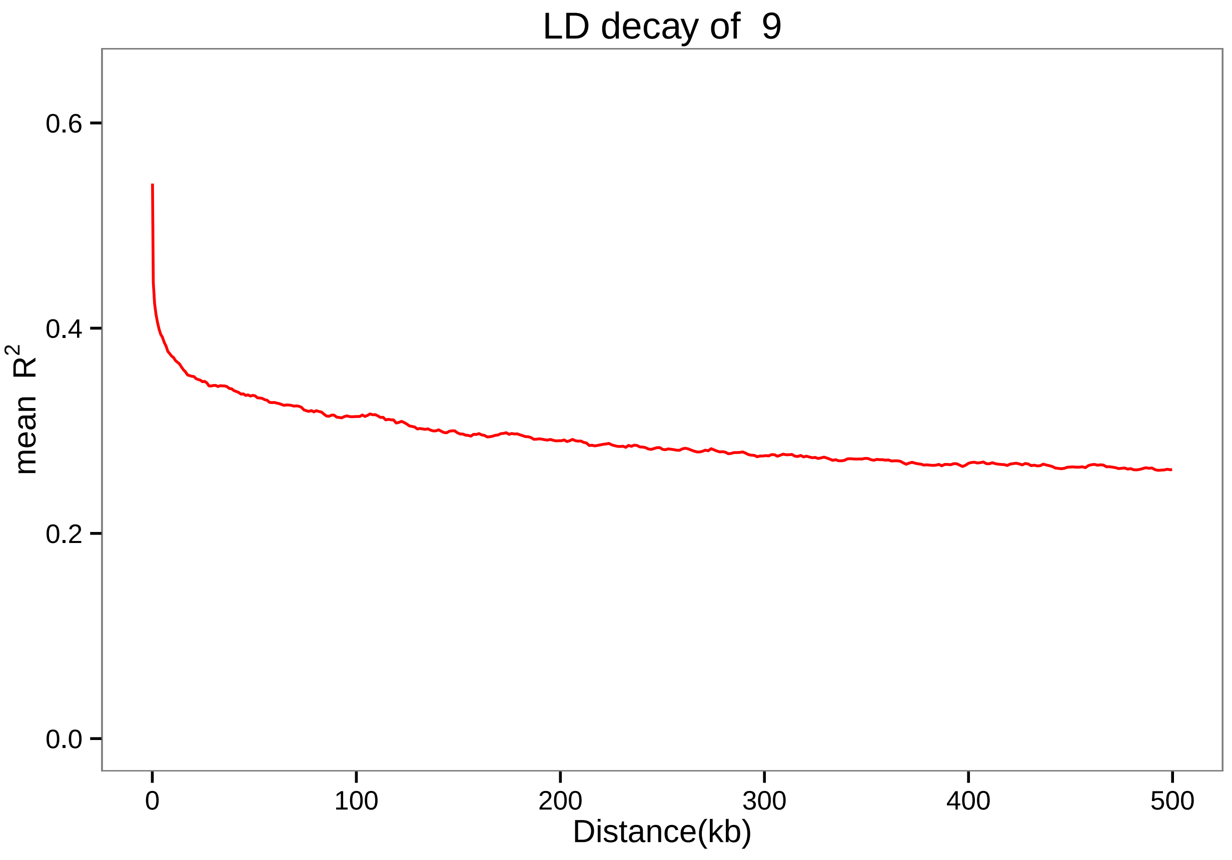
LD decay of 9
0.6
0.4
0.2
0.0
mean R2
0
100
200
300
400
500
Distance(kb)

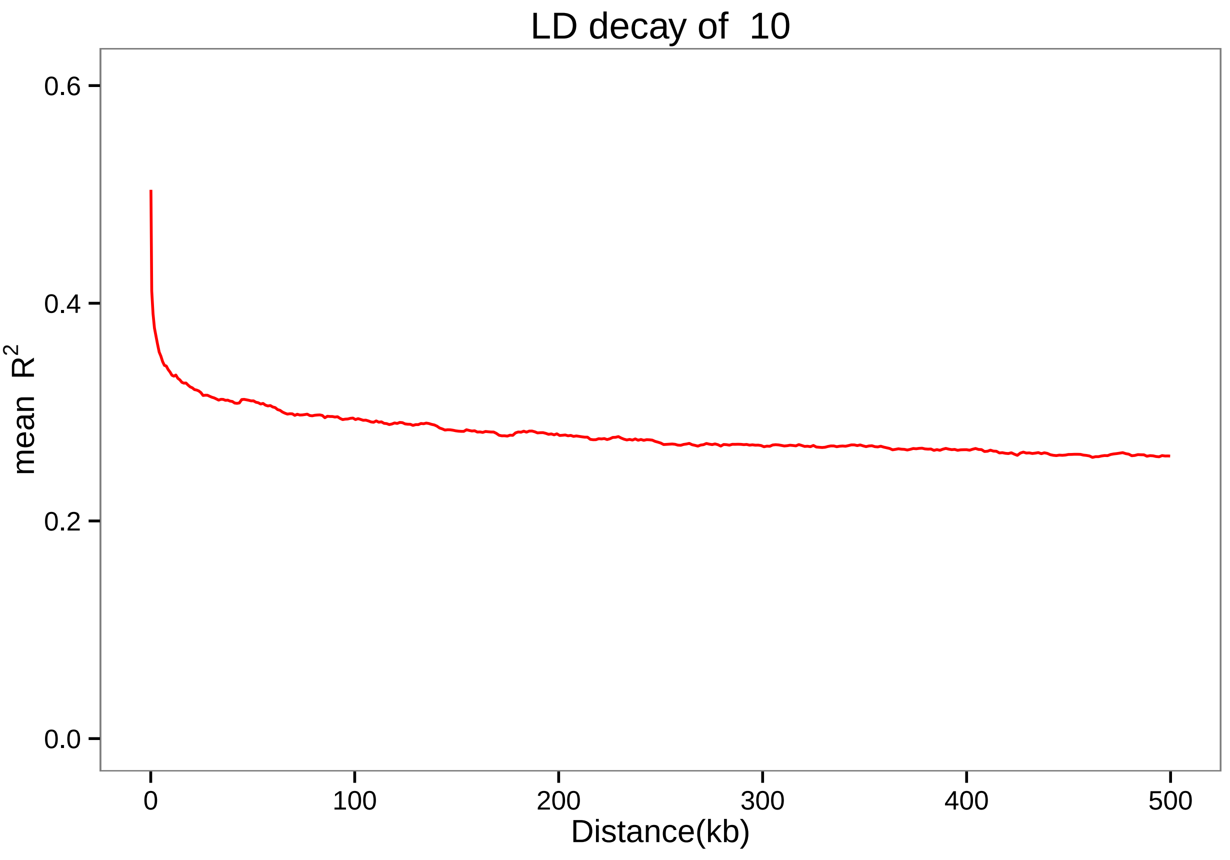

LD decay of 10
0.6
0.4
0.2
0.0
mean R^2
0
100
200
300
400
500
Distance(kb)

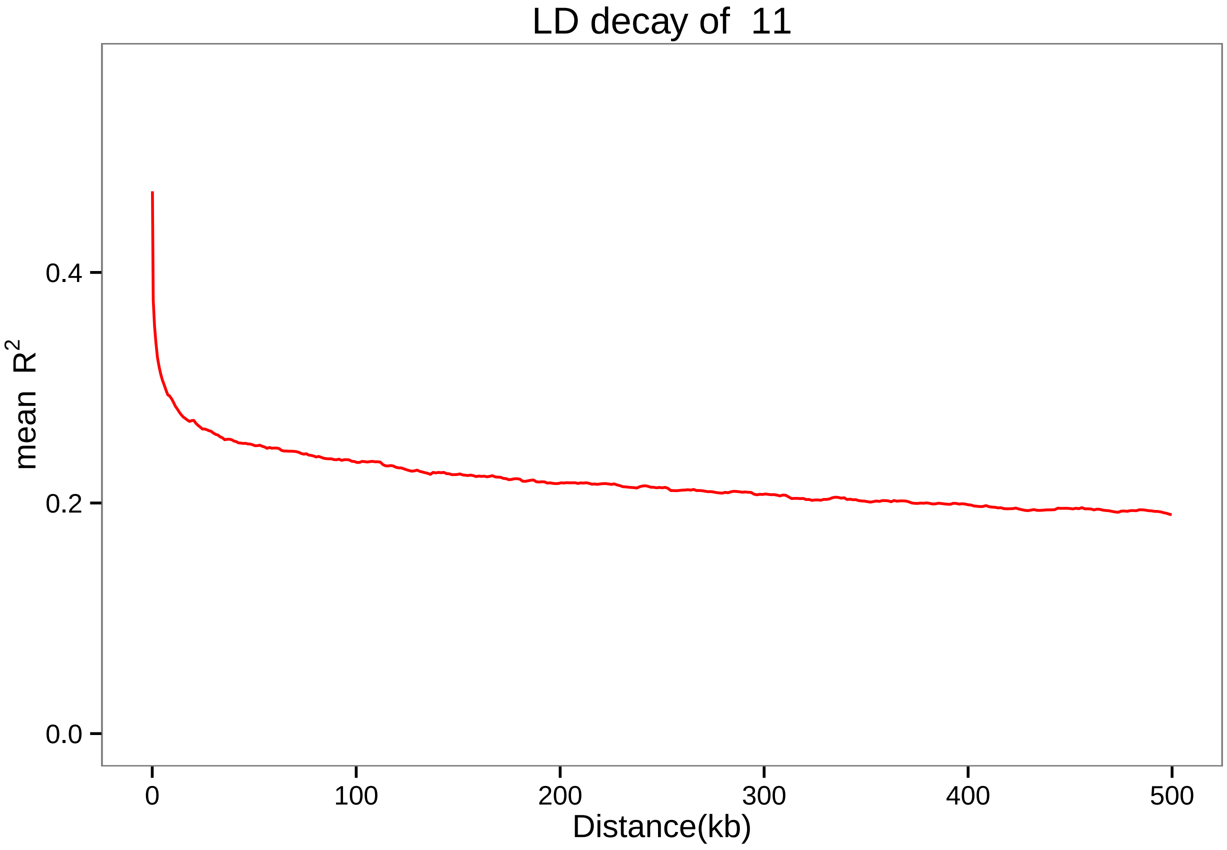

LD decay of 11
mean R^2
0.4
0.2
0.0
0
100
200
300
400
500
Distance(kb)

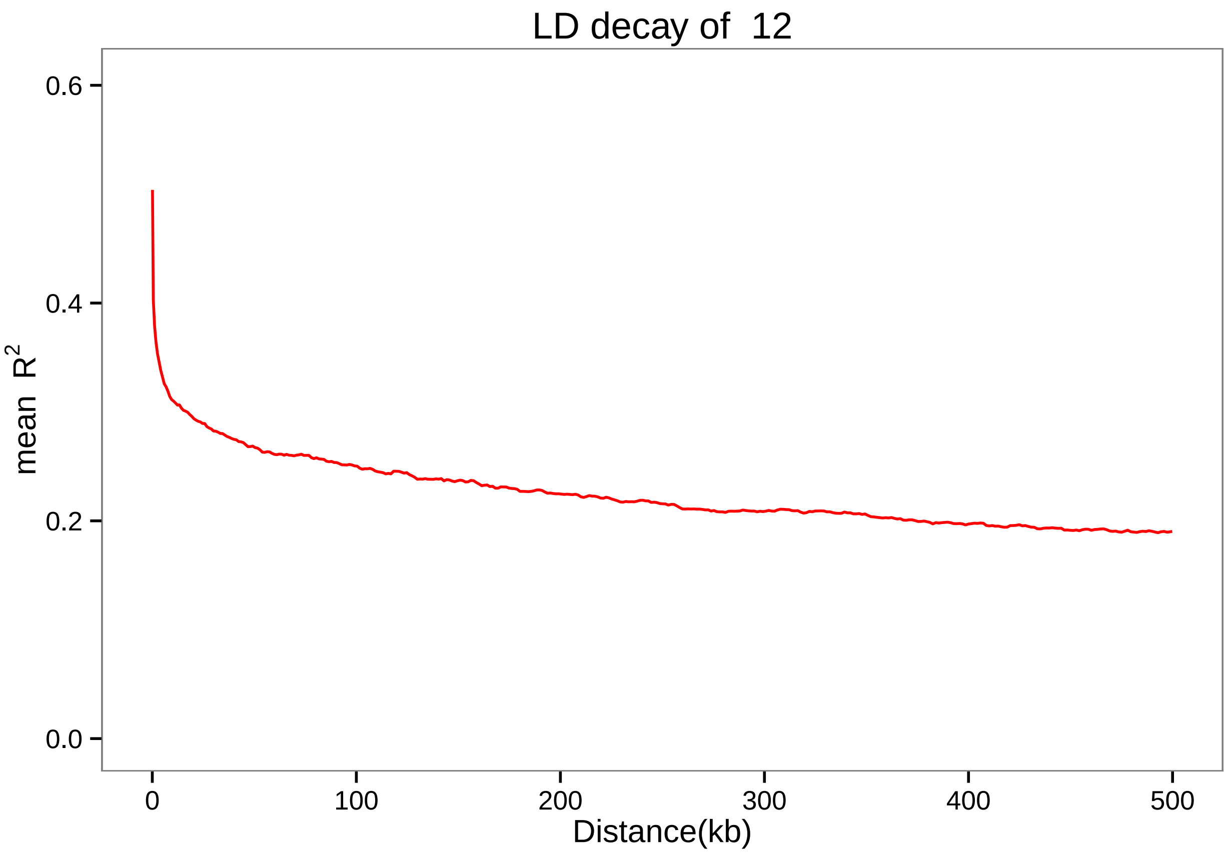
LD decay of 12
0.6
0.4
0.2
0.0
mean R2
0
100
200
300
400
500
Distance(kb)

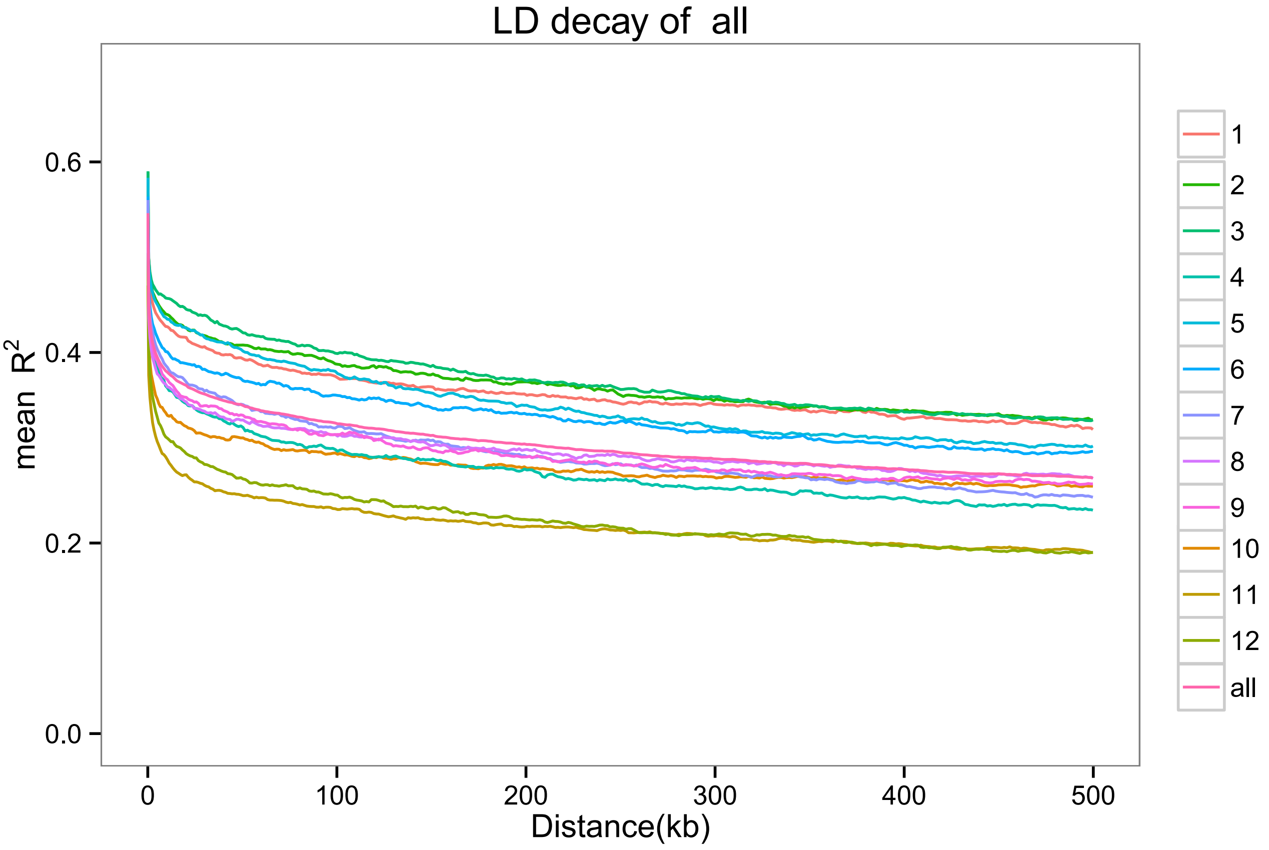
LD decay of all
mean R^2
0.0
0.2
0.4
0.6
0
100
200
300
400
500
Distance(kb)
1
2
3
4
5
6
7
8
9
10
11
12
all
