# Supplementary figures and images for: Using hyperspectral analysis as a potential high throughput phenotyping tool in GWAS for protein content of rice quality
Source: Plant Methods. 2019 May 23;15:54. doi: 10.1186/s13007-019-0432-x (PMC6532189; doi:10.1186/s13007-019-0432-x)

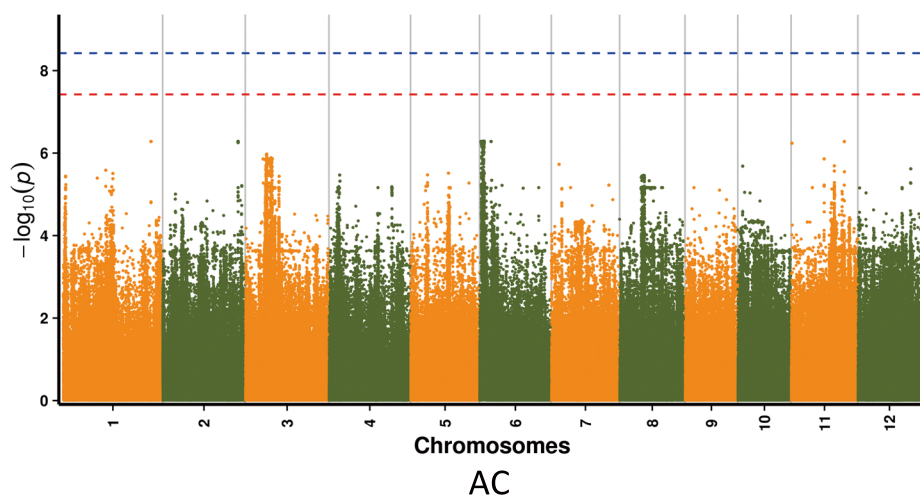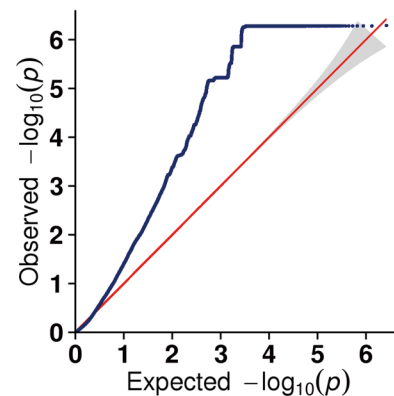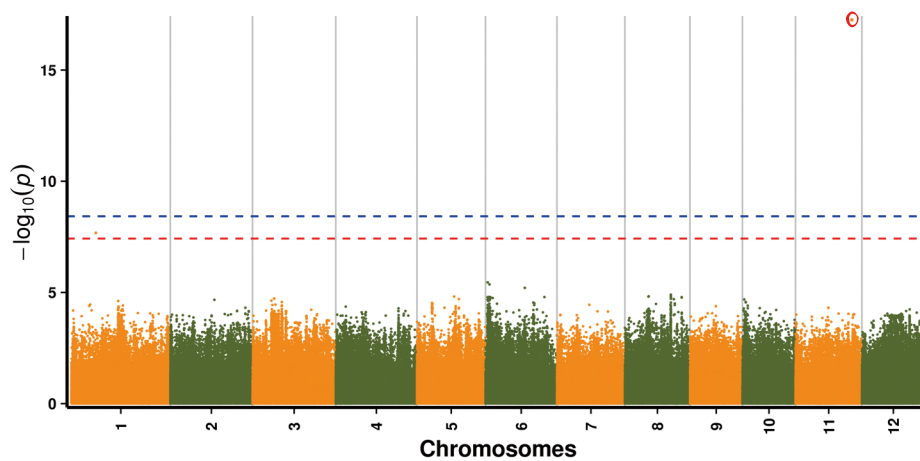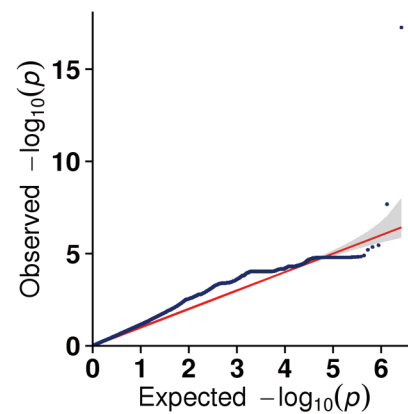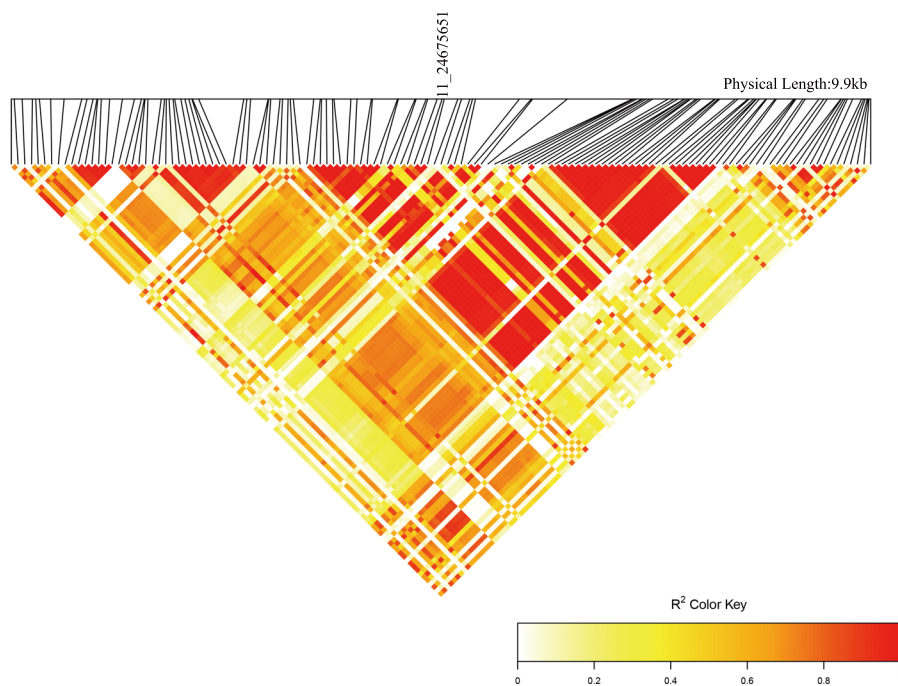

GC

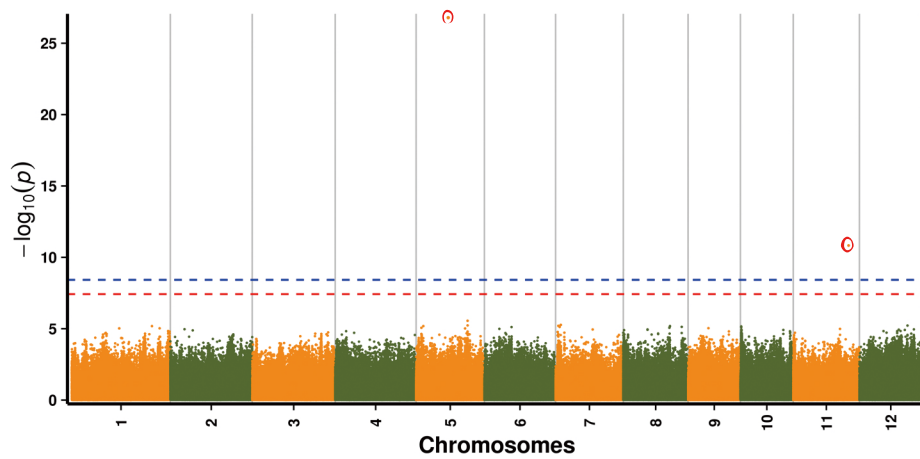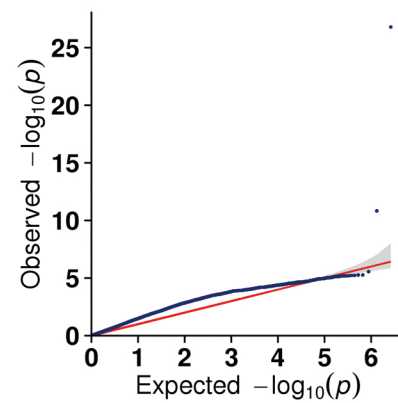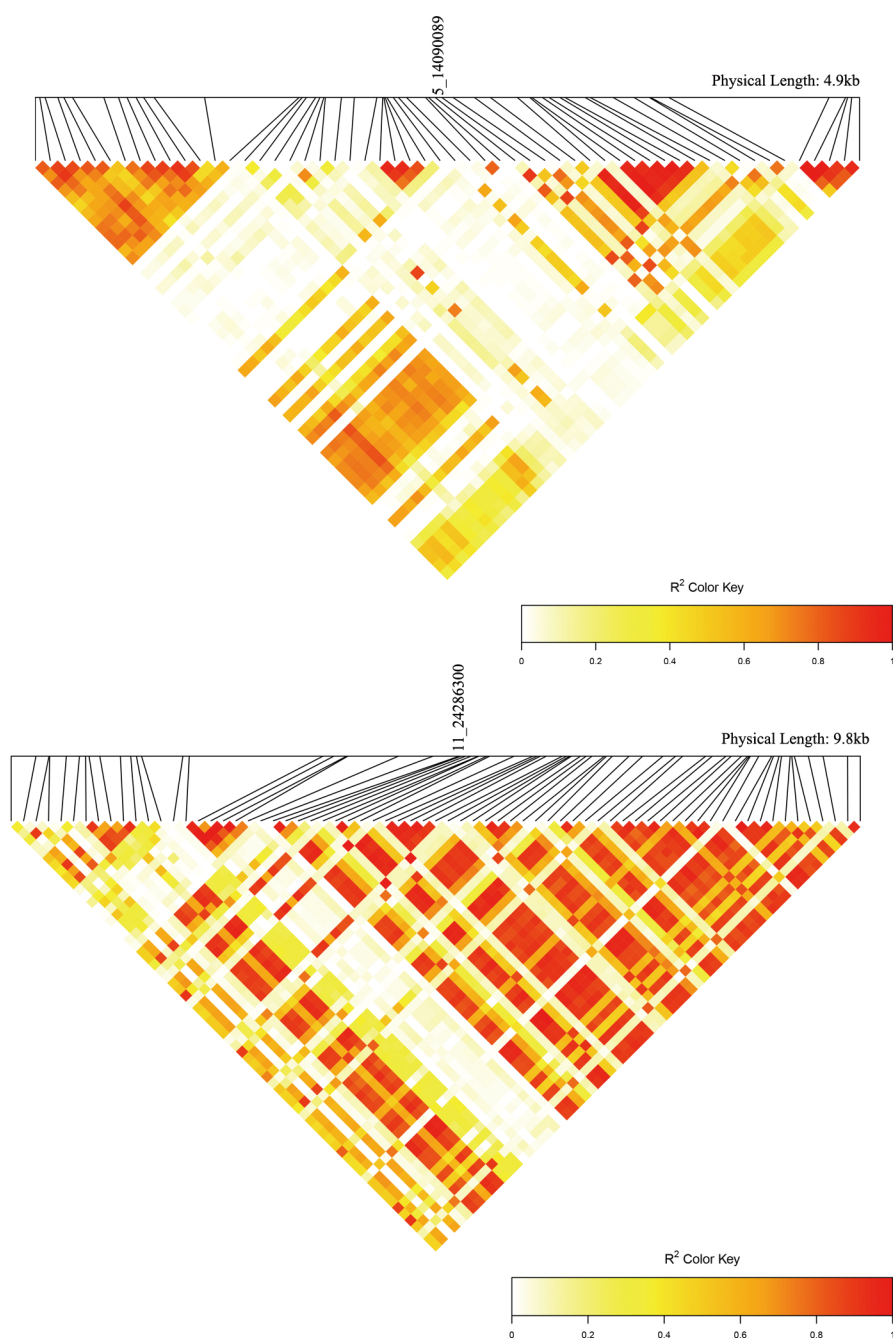

ASV

Supplement: Supplementary file 5 — Additional file 5: Fig. S3. GWAS results of other traits. [file 13007_2019_432_MOESM5_ESM.pdf]

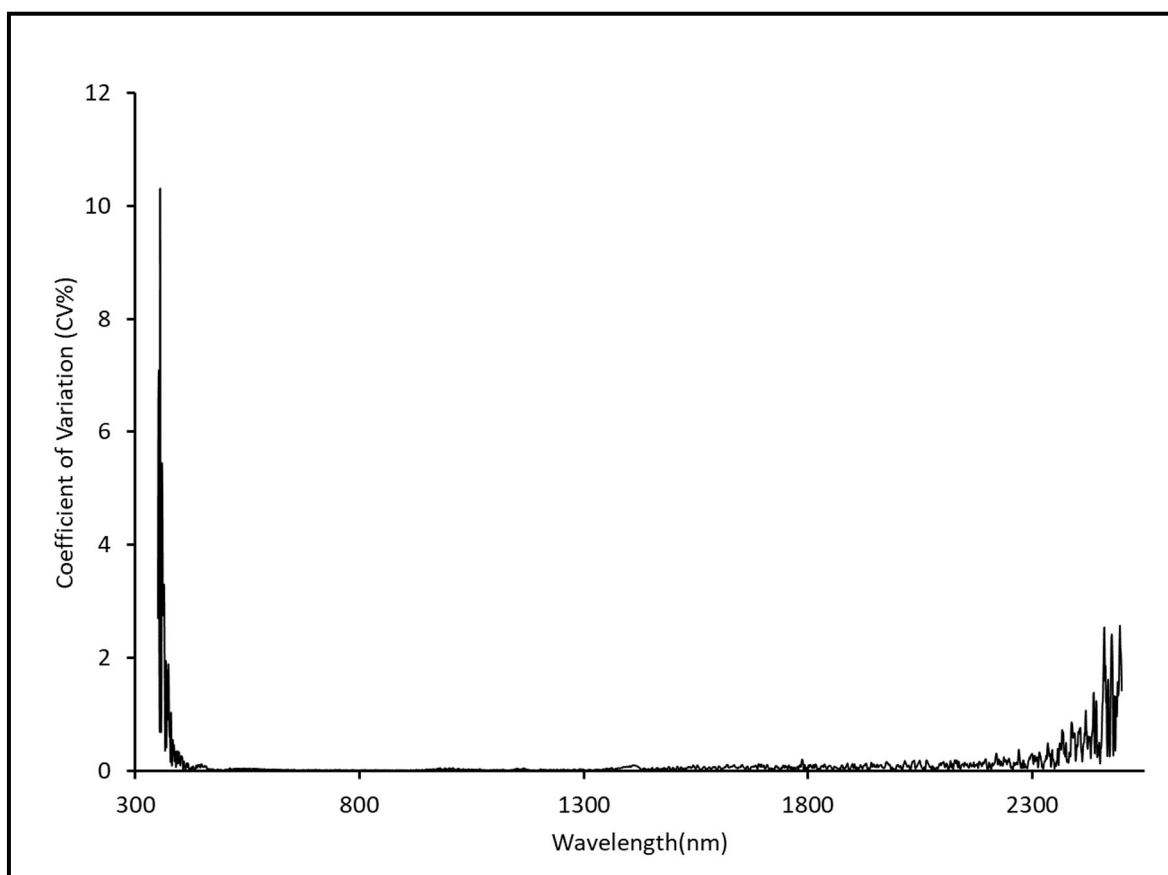

Fig. 4S The distribution of reflectance of all the spectrum based on the white reference

Supplement: Supplementary file 9 — Additional file 9: Fig. S4. Distribution of reflectance for signal-to-noise ratio. [file 13007_2019_432_MOESM9_ESM.pdf]

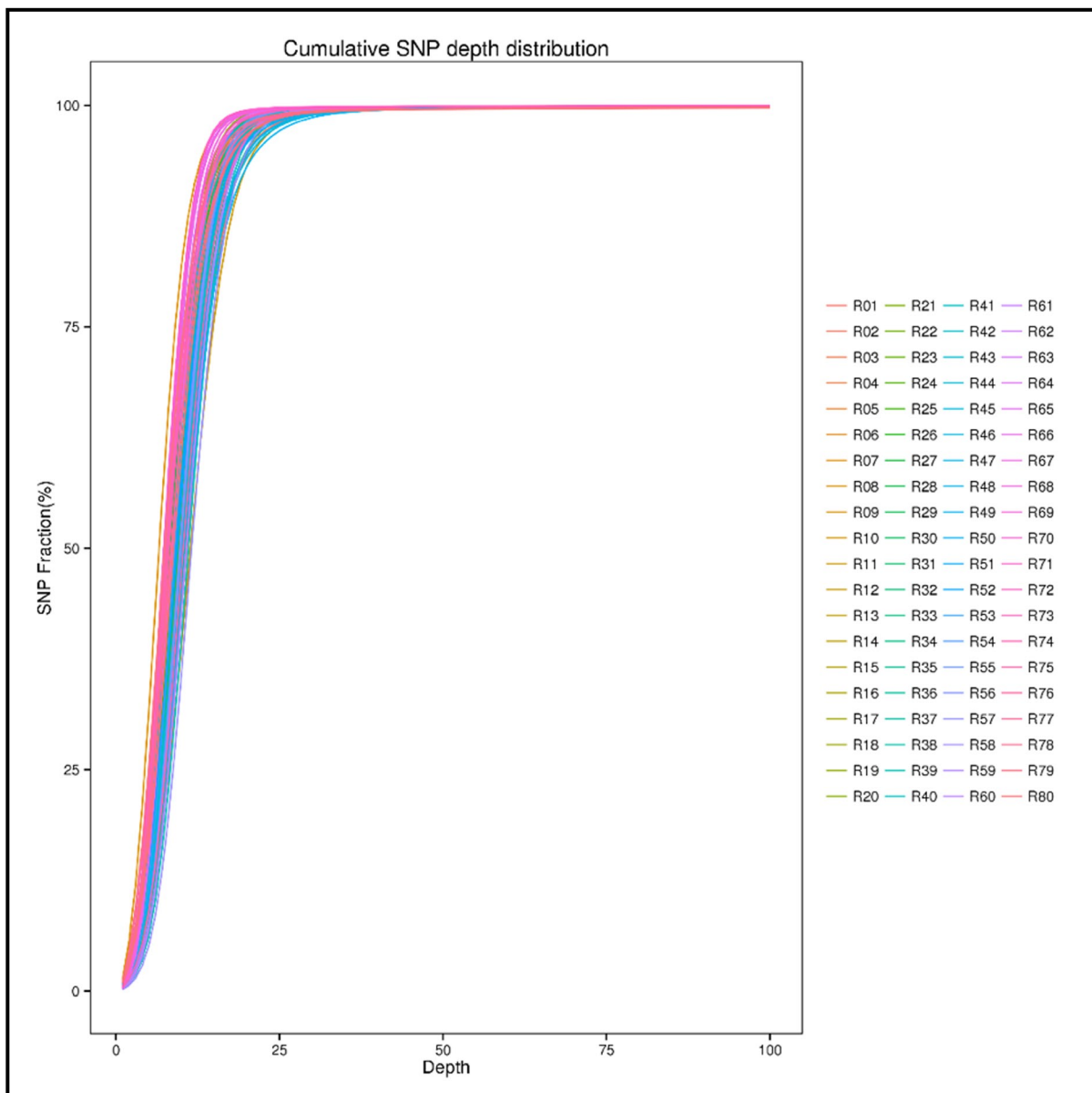

Fig. 5S The accumulative SNP depth distribution of all samples

Supplement: Supplementary file 10 — Additional file 10: Fig. S5. The distribution of accumulative SNP depth. [file 13007_2019_432_MOESM10_ESM.pdf]
